# A Boolean-based machine learning framework identifies predictive biomarkers of HSP90-targeted therapy response in prostate cancer

**DOI:** 10.3389/fmolb.2023.1094321

**Published:** 2023-01-19

**Authors:** Sung-Young Shin, Margaret M. Centenera, Joshua T. Hodgson, Elizabeth V. Nguyen, Lisa M. Butler, Roger J. Daly, Lan K. Nguyen

**Affiliations:** ^1^ Department of Biochemistry and Molecular Biology, Monash University, Clayton, VIC, Australia; ^2^ Cancer Program, Biomedicine Discovery Institute, Monash University, Clayton, VIC, Australia; ^3^ South Australian Immunogenomics Cancer Institute and Freemasons Foundation Centre for Men’s Health, University of Adelaide, Adelaide, SA, Australia; ^4^ South Australian Health and Medical Research Institute, Adelaide, SA, Australia

**Keywords:** precision oncology, predictive biomarker, machine learning, feature selection, Boolean function minimization, prostate cancer, Hsp90 inhibitor, 17-AAG

## Abstract

Precision medicine has emerged as an important paradigm in oncology, driven by the significant heterogeneity of individual patients’ tumour. A key prerequisite for effective implementation of precision oncology is the development of companion biomarkers that can predict response to anti-cancer therapies and guide patient selection for clinical trials and/or treatment. However, reliable predictive biomarkers are currently lacking for many anti-cancer therapies, hampering their clinical application. Here, we developed a novel machine learning-based framework to derive predictive multi-gene biomarker panels and associated expression signatures that accurately predict cancer drug sensitivity. We demonstrated the power of the approach by applying it to identify response biomarker panels for an Hsp90-based therapy in prostate cancer, using proteomic data profiled from prostate cancer patient-derived explants. Our approach employs a rational feature section strategy to maximise model performance, and innovatively utilizes Boolean algebra methods to derive specific expression signatures of the marker proteins. Given suitable data for model training, the approach is also applicable to other cancer drug agents in different tumour settings.

## Introduction

Precision treatment has become an important treatment modality in oncology, where the molecular makeup of patients’ tumour dictates therapeutic decisions. Identifying predictive biomarkers of treatment response that aid stratification of patients is critical for effective deployment of personalized oncology (Barretina et al., 2012; Geeleher et al., 2014; Nalejska et al., 2014). However, for most existing cancer drug agents including those that have been clinically approved (Pernas et al., 2018; Zhong et al., 2021), we currently lack companion predictive biomarkers that can reliably predict treatment response and inform patient selection. Thus, identification of predictive response biomarkers for cancer therapies represents a broad and unmet clinical need.

A major challenge that complicates the identification of response biomarkers is the multi-factorial determinant of cellular response to drug treatment, which is further accentuated by the extensive tumour heterogeneity between patients (Turajlic et al., 2019; Xi et al., 2019; Lee et al., 2021). Consequently, except for a few notable cases of clinical success (Quintás-Cardama and Cortes, 2009; Dieci et al., 2020), single-gene biomarkers are insufficient for predicting treatment responses and unlikely to be clinically useful (Nguyen et al., 2016). Instead, multi-gene biomarker panels are more likely to capture the complexity underpinning tumour drug response, and deliver better prediction (Zhu et al., 2011; Lima et al., 2019). Biomarker discovery approaches therefore should explicitly model combinations of relevant marker genes/proteins.

Computational methods have been key in the derivation of response biomarkers for cancer therapeutics (Menden et al., 2013; Tabl et al., 2019; Fortino et al., 2020). A simple but commonly used approach is to identify genes (or proteins) that are differentially expressed between treatment-sensitive and -resistant groups using–omics data such as transcriptomic or proteomic data (Chen et al., 2016; Nguyen et al., 2018). However, the degree of differential expression of a gene (based on fold-change and/or *p*-value) is not a good indicator of its predictive power towards treatment responsiveness. Moreover, the lists of DEGs are typically very long, and without further analysis to prioritize and narrow them down, the applicability of DEGs-based approaches remains limited. Recently, more sophisticated approaches such as machine learning (ML) have been applied to the biomarker discovery domain (Parca et al., 2019; Tabl et al., 2019; Fortino et al., 2020; Nguyen et al., 2021).

Yet, the ‘curse of dimensionality’ widespread in pharmacogenomics data - where the number of molecular features often far exceeds the number of biological samples - necessitates the development of feature selection strategies for ML algorithms (Huang et al., 2018; Nguyen et al., 2021; Ogunleye et al., 2022.). For example, Ballester and others have developed a scheme termed Optimal Model Complexity (OMC) aimed at identifying a smaller subset of informative features from the much larger original feature space, and integrated OMC with various ML algorithms (Bomane et al., 2019; Naulaerts et al., 2020; Nguyen et al., 2021). OMC works by ranking the features using the *p*-values obtained from univariate statistical tests to correlate between each feature and the corresponding labels (e.g., IC50 values of treated drugs), thereby pinpointing the most relevant features prior to model training (Nguyen et al., 2021). OMC-based XGBoost was employed to predict cancer drug response using pharmacogenomic data derived from either cancer cell lines (Yang et al., 2013; Naulaerts et al., 2020), or cancer patient-derived xenografts (Gao et al., 2015; Nguyen et al., 2021). In addition, Bomane et al. has also applied OMC to other ML algorithms, including Random Forest and LightGBM, to predict response to paclitaxel treatment in breast cancer (Bomane et al., 2019). On the other hand, Parca et al. (2019) selected potentially informative molecular genes for predicting cancer drug response by analysing the variance in gene expression profiles using cell lines based pharmacogenomic datasets. To predict cancer patient response to chemotherapeutic drugs, Huang et al. (2018) employed standard recursive feature elimination method to select for most relevant features (gene expression data) and applied it on top of support vector machine algorithm. Other studies utilise knowledge-based approaches to select likely relevant subsets of features: for example, by leveraging the protein-protein interaction network surrounding the drug targets (Kong et al., 2020), or restricting those to genes in the cancer gene census set (Futreal et al., 2004). However, due to lack of relevant patient-derived pharmacogenomic data, most ML studies to date have been performed using panels of cancer cell lines (Barretina et al., 2012; Garnett et al., 2012; Seashore-Ludlow et al., 2015; Iorio et al., 2016), which do not necessarily reflect the heterogeneity and drug sensitivity in human tumours (Borst and Wessels, 2010; Gillet et al., 2013).

**FIGURE 1:**
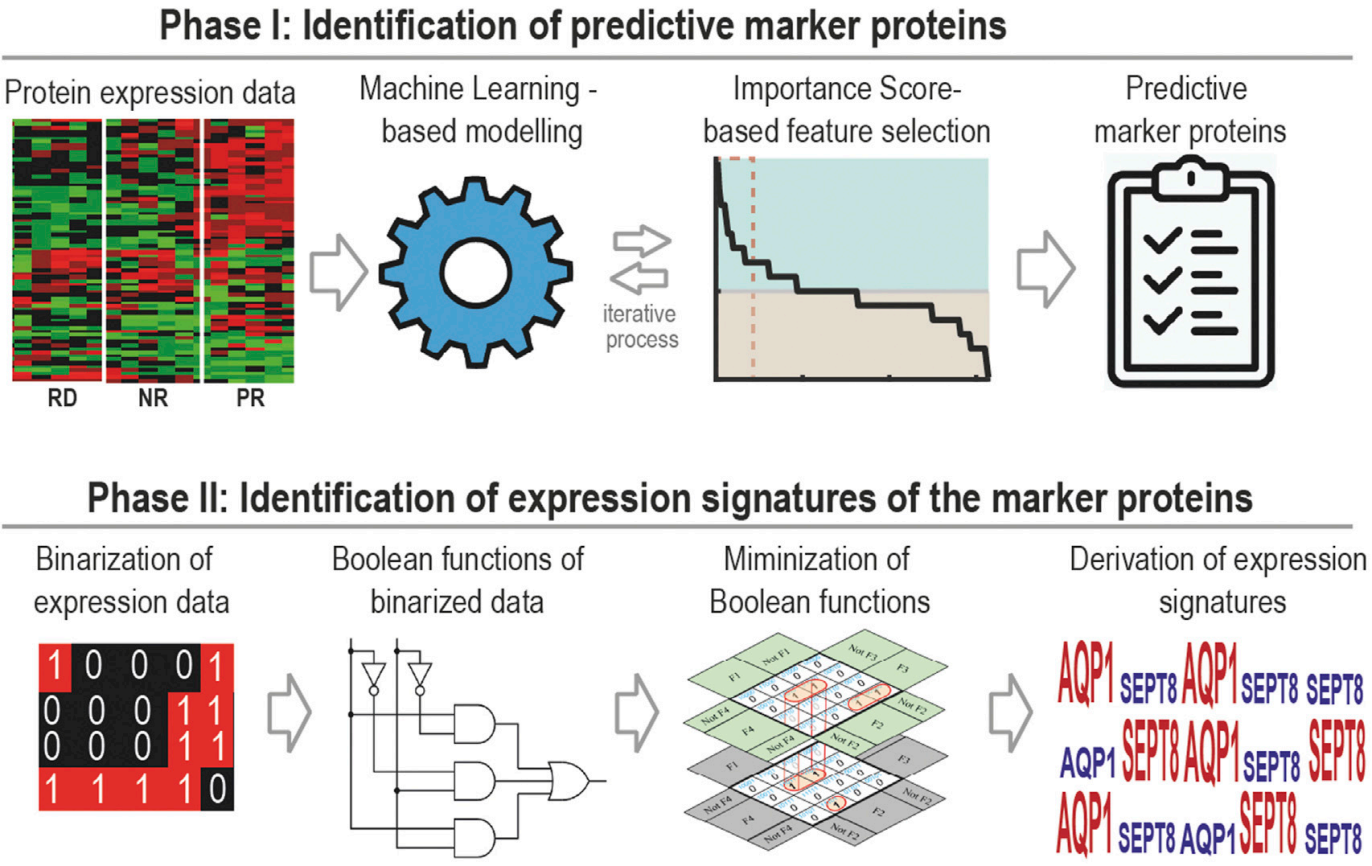
A general workflow of our two-phase computational framework, which couples supervised machine learning-based biomarker discovery with Boolean algebra-based signature derivation for the identification of predictive biomarkers.

In this study, we have developed a generally applicable machine learning framework for identification of multi-gene predictive biomarker panel and associated expression signatures for anti-cancer drugs. The approach comprises two phases (Figure 1). The first is to identify optimal biomarker panels that predict drug response using ML coupled with a new feature selection strategy. The second is to derive expression signatures of the identified biomarkers for different response groups utilizing a new Boolean function minimization-based pipeline. We applied the new approach to identify predictive biomarker panels and expression signatures for 17-AAG, a small-molecule inhibitor targeting heat shock protein 90 (Hsp90), using pharmacoproteomic data obtained from prostate cancer patient-derived explants (PDEs) (Cardillo and Ippoliti, 2006). Blocking Hsp90 is considered as an attractive therapeutic strategy for prostate cancer. This is because Hsp90 is commonly overexpressed in prostate cancer compared to normal prostate cells (Cardillo and Ippoliti, 2006); prostate cancer cells are selectively sensitive to Hsp90-directed agents; and Hsp90 clients include the androgen receptor (Trepel et al., 2010), a major driver of prostate tumorigenesis. However, despite the anti-tumour activity of Hsp90 inhibitors (e.g., 17-AAG) in preclinical models (Solit et al., 2002), the lack of companion predictive biomarkers for rational patient stratification have in part contributed to the poor response rates to these agents seen in clinical trials (Heath et al., 2008).

We derived a 16-protein biomarker panel that achieved 92% response prediction accuracy to 17-AAG. To facilitate clinical translation, we further reduced this to a compact 5-protein panel having 80% prediction accuracy, and identified associated expression signatures. Interrogation of prostate cancer patient data identified almost half of the patients with matching expression signatures, who may benefit from 17-AAG treatment. Overall, this work presents a novel ML framework that aids the discovery of predictive biomarker panels for improved patient selection and treatment of cancer.

## Materials and methods

### Patient data analysis

Patient data from two prostate cancer patient cohorts were used to interrogate the utility of our derived 5-gene biomarker panel as a potential patient stratification tool. These include the TCGA (Pancancer Atlas, (Hoadley et al., 2018)) cohort containing 494 patients; and another independent prostate cancer patient (PNAS 2019, (Abida et al., 2019)) cohort containing 208 patients. Patient-specific transcriptomic data was downloaded from the cBioPortal for Cancer Genomics database (Cerami et al., 2012; Gao et al., 2013)) for analysis (see Figure 5E, left panels).

### Patient-derived explant (PDE)

Fresh prostate cancer specimens were obtained with written informed consent through the Australian Prostate Cancer BioResource from men undergoing robotic radical prostatectomy at the Royal Adelaide Hospital and St Andrew’s Hospital (Adelaide, South Australia). Tumors from two cohorts of patients were used for this study: a discovery cohort (*n* = 40, obtained from (Nguyen et al., 2018)), and a test cohort (n = 7). A single 6 mm core of tissue was obtained per patient. A longitudinal section of the entire core was taken for hematoxylin and eosin (H&E) analysis of tumor content. The remaining tissue was dissected into 1 mm3 pieces and cultured in triplicate on a presoaked gelatin sponge (Johnson and Johnson, New Brunswick, NJ) in 24-well plates containing 500 L RPMI 1640 with 10% FBS, 1 antibiotic/antimycotic solution (Sigma, St Louis, MO), 0.01 mg/ml hydrocortisone, 0.01 mg/ml insulin (Sigma) and cultured for 48 h with 17-AAG (500 nM) or DMSO vehicle alone as previously described (Nguyen et al., 2018). Mass spectrometry-based proteomic profiling were performed on the discovery cohort as described in (Nguyen et al., 2018), and these data were used as inputs (features) for our ML models. Treatment response was quantified based on the relative expression of the proliferative marker Ki67, measured post drug treatment by immunohistochemical (IHC) assay (Nguyen et al., 2018).

### Immunohistochemical staining

Paraffin-embedded tissues were sectioned (2 mM) on Ultraplus slides prior to H&E staining and IHC detection of Ki67 (Agilent, M7240 antibody; 1:200 dilution, Santa Clara, CA). IHC staining was performed and tissues assessed for tumor content and Ki67 positivity in a blinded fashion as described in (Armstrong et al., 2016).

### qRT-PCR

Real-Time Quantitative Reverse Transcription PCR was used to measure the baseline expression of the five genes identified in our 5-gene biomarker panel: AQP1, SEPT8, RBM17, TRIM47, and VPS25 for the testing PDE cohort (Supplementary Table S5). qRT-PCR was also used to measure the baseline and post-treatment expression of *MKi-67*, the gene encoding of the proliferative marker Ki67. Cultured patient derived explants were placed in a Precellys Tissue Homogenizer (Bertin instruments) for 2 cycles at 6500rpm. RNA was extracted from tissue homogenate using miRNeasy mini kit (Qiagen) according to manufactures instructions. RNA (700 ng) was reversed transcribed to cDNA using IScript cDNA synthesis kit (Bio-Rad). QRT-PCR was performed with a 1:10 dilution of cDNA using SYBR green (Bio-Rad) on a CFX 384 real time system (Bio-Rad). Relative gene expression was calculated using the comparative ct method and normalized to internal control genes GAPDH & TUBA1B. Primer sequences used for PCR are given in Supplementary Table S5.

### ML implementation

To classify drug response groups, we developed a multi-class Support Vector Machine (SVM) and an artificial neural network (ANN) classifier using the MATLAB function *fitcecoc* and *patternnet*, respectively. For the multi-class SVM model, we set the “standardized” option to “true,” which normalized the predictor data and used the option *linear* as the kernel function of mSVM. For the ANN model, the predictor data was also normalized, and the size of the hidden layers was set to 10. Protein expression data profiled from 40 prostate cancer PDE samples was used for model training and testing. For training and testing, the functions’ default settings were used (e.g., scaled conjugate gradient backpropagation algorithm (Møller, 1993), implemented using MATLAB function *trainscg*), with 80%–20% data split ratio. For the implementation of K-Nearest Neighbor, Naïve Bayes, Random Forest, and AdaBoost we used Matlab functions *fitcknn* (Distance = ‘Euclidean’), *fitcnb* (Kernel = ‘Normal’), *fitrensemble* (Method = ‘Bag’), *fitcensemble* (Method = ‘AdaBoostM2’), respectively. For Deep Forest, we utilized the Matlab codes developed by (Zhou and Feng, 2017), available at Github at https://github.com/cnzakimuena/casForest.git.

These data were deposited onto the Mass spectrometry Interactive Virtual Environment (www.massive.ucsd.edu) with identifier: MSV000082244 (Nguyen et al., 2018). Model validation was performed using the function *predict* for mSVM and *sim* for ANN, respectively. ROC curves and confusion table were generated using functions *roc* and *confusionchart* in MATLAB. All the relevant codes were deposited on Github at https://github.com/NguyenLabNetworkModeling/GFFS-Biomarker.

### Importance score calculation

The importance score (IS) associated with a feature was calculated through performing a systematic ‘feature drop-out’ analysis. For this, each feature (e.g., DEP) was removed from the feature list, one at a time, and the effect on model prediction performance was assessed. IS measures the difference in prediction accuracy between the ‘drop-out’ and the original mSVM model, computed as follows:

$$IS\left(i\right)=-\frac{{{PA}_{i}-PA}_{O}}{{PA}_{O}}$$ (1)

where PA_*O*_ and PA_*i*_ represent the prediction accuracy of the original mSVM and the “drop-out” model where input feature *i* is removed from the feature list. Thus, IS > 0, <0, = 0 indicate the dropped-out feature has a positive, negative, or no impact on the model predictive performance, respectively.

### Feature selection

Our feature selection strategy GFFS was implemented based on the IS values, as described in the text. To compare different feature selection algorithms, we implemented ReliefF using the function *relief* and MRMR using the function *fscmrmr* in MATLAB. For LASSO regression, we used the function *fitcecoc* and *lasso* as a regularization method. To calculate the importance score of the Boosting and Bagging ensemble models, we used the functions predictorImportance and oobPermutedPredictorImportance in MATLAB. We also implemented RFE and FFE strategies on top of the SVM.

### Explainable ML analyses

SHAP and LIME analyses were implemented using the *shapley* and *lime* functions in MATLAB, and *AdaBoostM2* as a ‘black-box ensemble model’.

### Boolean functions and minimization

A Boolean function is an algebraic expression consisting of *n*-binary variables*, f* (*x*_1_, *x*_2_, … , *x*_n_). Boolean functions can be formulated through Sum of Product (SOP) or Product of Sum (POS). In SOP, different product terms of inputs are summed together, where the products are logical AND the sum are OR operators. For example: *x*′+*xy* + *yz*’ where *x*, *y* and *z* are binary variables and prime (‘) represent complement of a variable, that is if x = 0 then *x*′ = 1. On the other hand, in POS products of different summation terms of inputs are taken, e.g. (*x*′)⋅(*x* + *y*)⋅(*y* + *z*′). Boolean functions can be simplified using Boolean laws and theorems (Hanf, 1975; Whitesitt, 2012). The process of simplifying the algebraic expression of a Boolean function is called ‘minimization’. To minimize the Boolean function, we employed the Quinine-MacCluskey algorithm (Jain et al., 2008) implemented in MATLAB (http://www.tu-harburg.de/∼rtsap/#Programs).

### Statistical and bioinformatic analysis

Statistical t-tests were performed using GraphPad Prism 9 and Matlab R2022b. For the KEGG pathway and the GO function analysis we utilized Enrichr web application (Kuleshov et al., 2016), which can be accessed at https://maayanlab.cloud/Enrichr/.

### Pharmacoproteomic data from prostate cancer PDEs for machine learning

**FIGURE 2:**
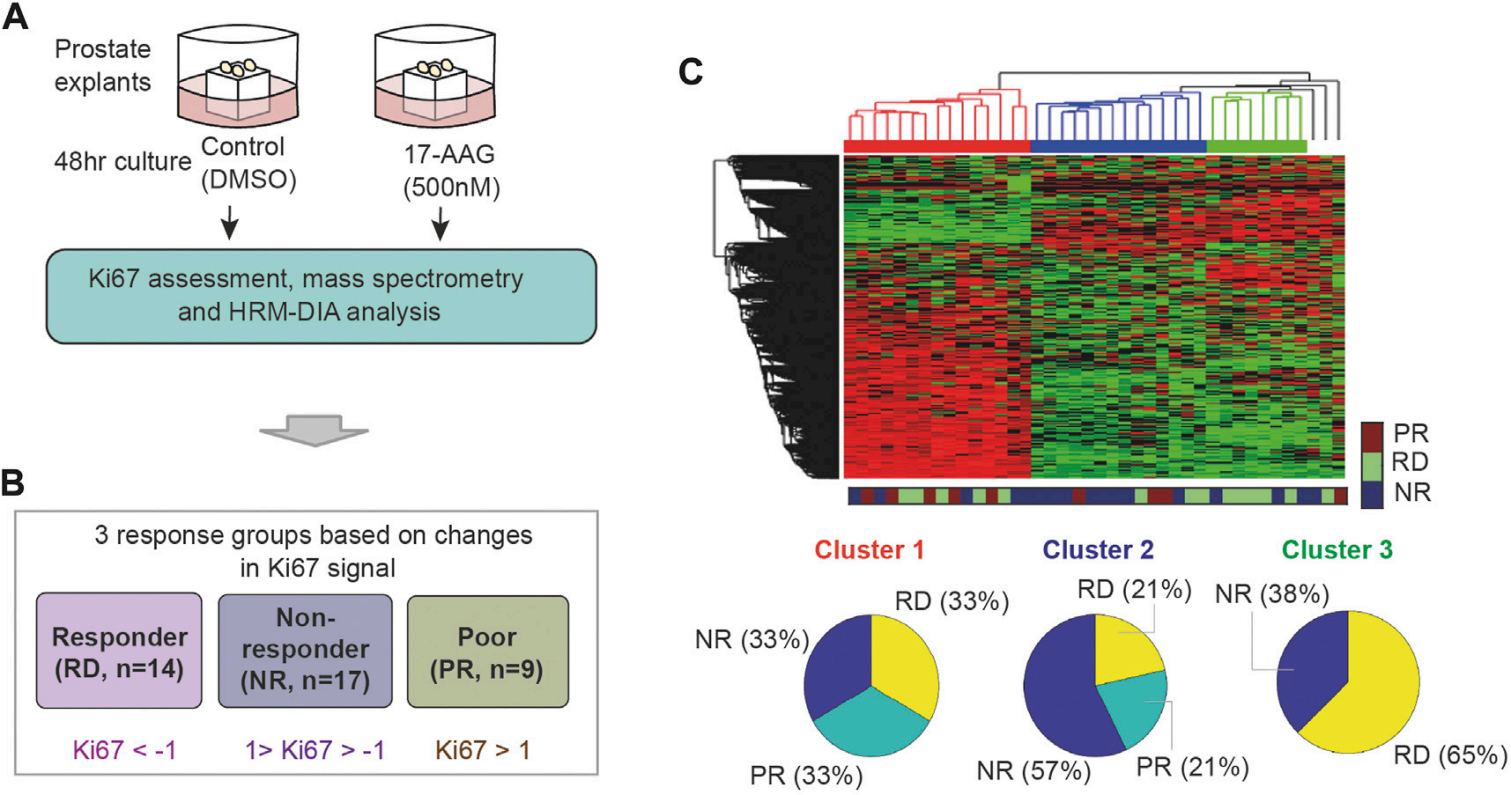
Unsupervised clustering of the PDEs’ response to 17-AAG treatment. **(A)** Pharmacoproteomic data obtained from 40 prostate cancer PDEs (Nguyen et al., 2018). **(B)** Classification of the PDEs into three response groups based on Ki67 positivity post 17-AAG treatment: RD, PR, and NR with indicated fold-change in Ki67 positivity (in log2 scale). **(C)** Unsupervised hierarchical clustering of the PDEs using the proteomic data, which failed to reasonably predict response to 17-AAG.

To recapitulate the *in vivo* response of prostate cancer to therapies, we have previously developed an *ex vivo* culturing model of prostate cancer tissue that retains the structure and stromal-epithelial interactions of the tumor microenvironment and provides the level of disease heterogeneity seen in patients (Centenera et al., 2012). Using this system, we established in a previous study 40 prostate cancer patient-derived explants (PDEs) and subjected them to either vehicle (DMSO) or 17-AAG (500 nM) treatment for 48 h (Figure 2A, see also Materials and Methods) (Nguyen et al., 2018). Treatment response was quantified based on the relative expression of the proliferative marker Ki67, measured post drug treatment by immunohistochemical assay (Nguyen et al., 2018). In addition, we performed mass spectrometry-based proteomic profiling and HRM-DIA data analysis on the corresponding 40 PDEs, which identified the expression of 3,766 quantifiable proteins prior to 17-AAG treatment (Nguyen et al., 2018). These datasets will be used in this study to develop companion biomarkers that accurately predict response to 17-AAG treatment.

To label the data, the PDE samples were classified into three distinct response groups based on changes in Ki67 positivity upon treatment with 17-AAG (Nguyen et al., 2018). These are depicted in Figure 2B: (i) RD (responders) group containing PDEs having > two-fold decrease in Ki67 positivity; (ii) PR (poor responders) group containing PDEs with < two-fold increase in Ki67 positivity; and (iii) NR (non-responders) group with Ki67 positivity in between. As a result, 14 PDEs were classified as RD, 17 as NR and 9 as PR (Figure 2B; Supplementary Table S2). Of note, the PDE proteomic data has 0.16% missing (undetectable) values and they were imputed with random values generated from a uniform distribution between 0 and 1 (1 is the minimal machine-detectable protein amount) (Wei et al., 2018). Together, the PDE data consists of protein expression levels of 3,766 proteins serving as ‘input features’ and Ki67-based response classification serving as ‘labelled outputs’ for development of ML models.

## Results

### Supervised ML using differentially expressed proteins (DEPs) sub-optimally predicts 17-AAG response

Using expression levels of all the 3,766 proteins as inputs, we first tested whether unsupervised hierarchical clustering could predict the PDE response to 17-AAG treatment. While this identified three distinct clusters, they poorly reflected the labelled response groups (Figure 2C). Each of the three clusters comprises a good mixture of RD, NR and PR samples, suggesting that unsupervised clustering could not reasonably predict response to 17-AAG.

**FIGURE 3:**
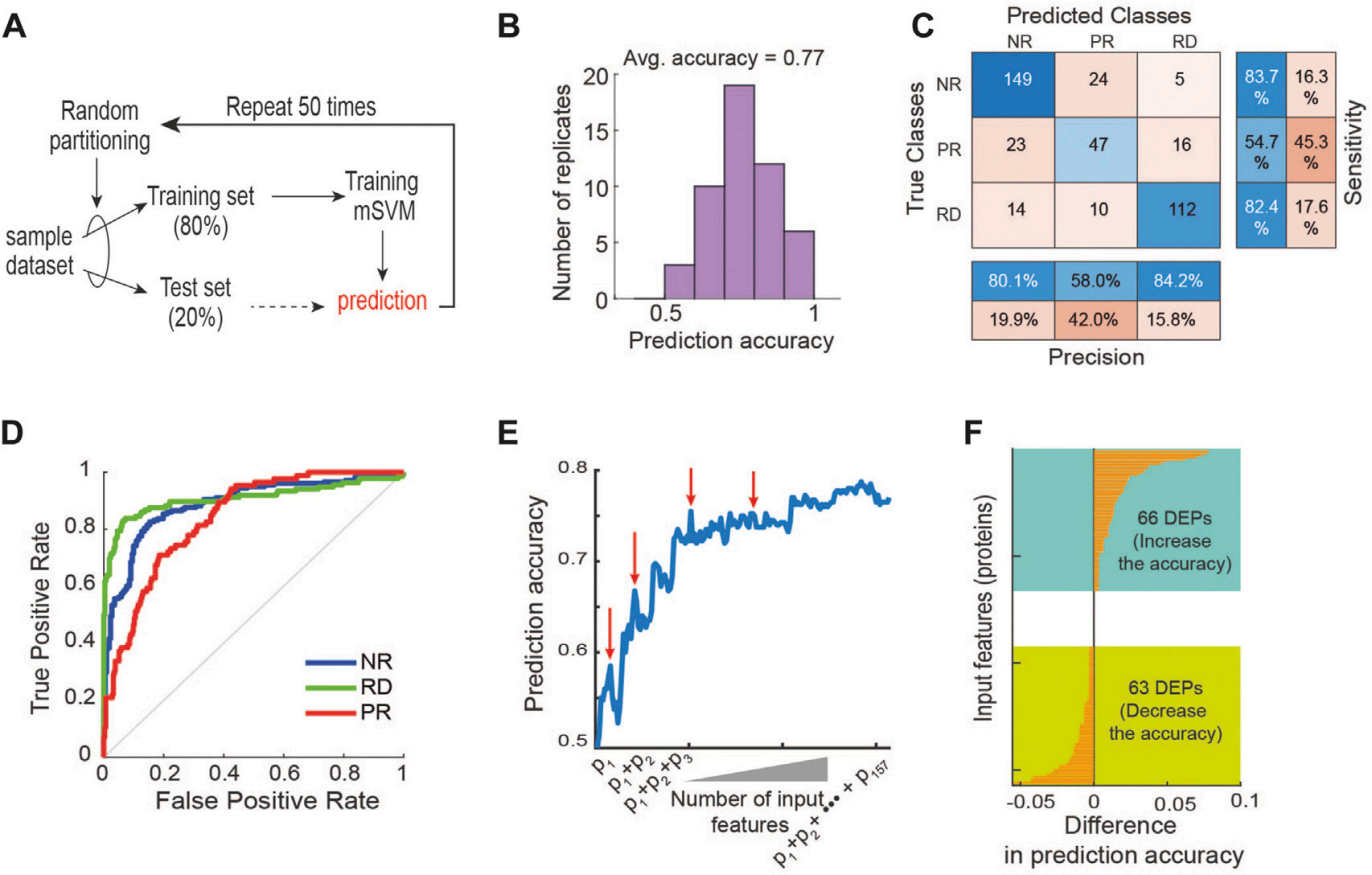
Machine learning-based prediction of response to 17-AAG. **(A)** A machine learning pipeline utilizing the repeated holdout method for training and testing with an mSVM model (see Materials and Methods). **(B)** Distribution of prediction accuracy performance across 50 model replicate runs. **(C)** Performance of the mSVM classifier using all 157 DEPs as input features, summarized in a confusion matrix. **(D)** Receiver operating characteristic (ROC) curves of the mSVM classifier for the RD, NR and PR response groups. **(E)** Impact of input feature space modulation on model prediction accuracy. Adding specific input features may worsen model performance, indicated by the red arrows. **(F)** Difference in the prediction accuracy when a new input feature is added to the training data.

Next, to examine if supervised ML methods would improve the response prediction, we developed a multi-class support vector machine (mSVM) model using the protein expression as inputs and the labelled drug responses (RD, PR, NR) as outputs (Figure 3A). The dataset was randomly divided into a training (80%, 32 PDEs) and a test set (20%, 8 PDEs). To avoid biases in data splitting and mitigate model overfitting, we held out the test set and trained the model with the training set. This training and test process were repeated 50 times to obtain reliable and robust performance evaluation (). We found that the model displayed an average prediction accuracy of ∼39% (Supplementary Figure S1). This poor performance is somewhat expected because the number of input variables/features (3,766) greatly exceeds the number of samples (40), a phenomenon known as ‘curse of dimensionality’ in ML (Hughes, 1968). By this principle, the prediction power of a ML classifier typically improves as the number of the features gradually increases, but after an threshold (i.e., optimal) number of features, adding more starts to diminish the model performance (Hughes, 1968). This is because the high dimensionality of the input data causes every observation to appear equidistant from the others, preventing meaningful clustering (Hughes, 1968). Moreover, irrelevant or partially relevant features can negatively impact model performance (John et al., 1994).

In order to circumvent the curse of dimensionality, we carried out a feature selection strategy with the goal to rationally reduce the number of non-relevant features (Cai et al., 2018; Gopika and Meena Kowshalya, 2018). As differentially expressed proteins (DEPs) often provide a good starting point for identifying potential biomarkers (Chen et al., 2016; Nguyen et al., 2018), we first performed differential expression analysis between the three response groups using analysis of variance (ANOVA) tests, and obtained a total of 157 DEPs (*p*-value <0.05, Supplementary Table S2). Unsupervised hierarchical clustering using these DEPs still failed to appropriately cluster the PDE samples (Supplementary Table S2), confirming the suboptimal performance of this approach. Next, we retrained the mSVM model using the 157 DEPs as input features and found that it displayed an average prediction accuracy of 77% (Figure 3B). Examining the confusion matrix results further showed that while the precision and sensitivity for the RD and NR groups are around and above 80%, they are below 55% for the PR group (Figure 3C), demonstrating the mSVM model did not perform well against the PR group. Consistently, analysis of the receiver operating characteristic (ROC) curves confirms that model performance against the PR group was inferior compared to the other groups (Figure 3D).

In addition to mSVM, for comparison purposes we also performed similar analyses using an array of common ML methods, including artificial neural network (ANN), K-Nearest Neighbor (KNN) (Min-Ling and Zhi-Hua, 2005; Zhang, 2016), Naive Bayes (Yousef et al., 2007), Decision Tree (Navada et al., 2011), AdaBoost (Feng et al., 2020), Random Forest and Deep Forest (Liu et al., 2012; Zhou and Feng, 2018; Su et al., 2019). The results show that mSVM was the best-performing algorithm, followed by ANN and KNN (Supplementary Figure S3). Like mSVM, the ANN model performed relatively poorly in predicting the PR group (Supplementary Figure S4). Together, these results suggest that although supervised ML approaches perform better than unsupervised hierarchical clustering, using all the DEPs as features may be inadequate for optimizing predictive power. This may be due to the noise exhibited by certain DEPs that bear no relevance in predicting response to 17-AAG, which interferes with the predictive signals from the relevant features, thereby lowering the model’s overall predictive performance (Blum and Langley, 1997).

To interrogate how modulation of the input feature space may influence performance of the mSVM, we systematically increased the number of features by adding the DEPs one by one to the training set and re-evaluated the model prediction accuracy. Figure 3E shows an overall upward trajectory of prediction accuracy as the number of feature increases. However, there were specific DEPs whose addition to the feature space actually worsened the model’s predictive power, evidenced by drops in the trajectory (indicated by red arrows, Figure 3E). Specifically, 66 of the 157 DEPs contributed positively to the model performance while 63 contributed negatively, and some had negligible effects on performance (Figure 3F). These results support the idea that irrelevant features can negatively impact the model’s ability to predict drug response, and thus rational selection of informative features is key in improving predictive performance.

### A novel ML framework maximises prediction accuracy through rational feature selection

**FIGURE 4:**
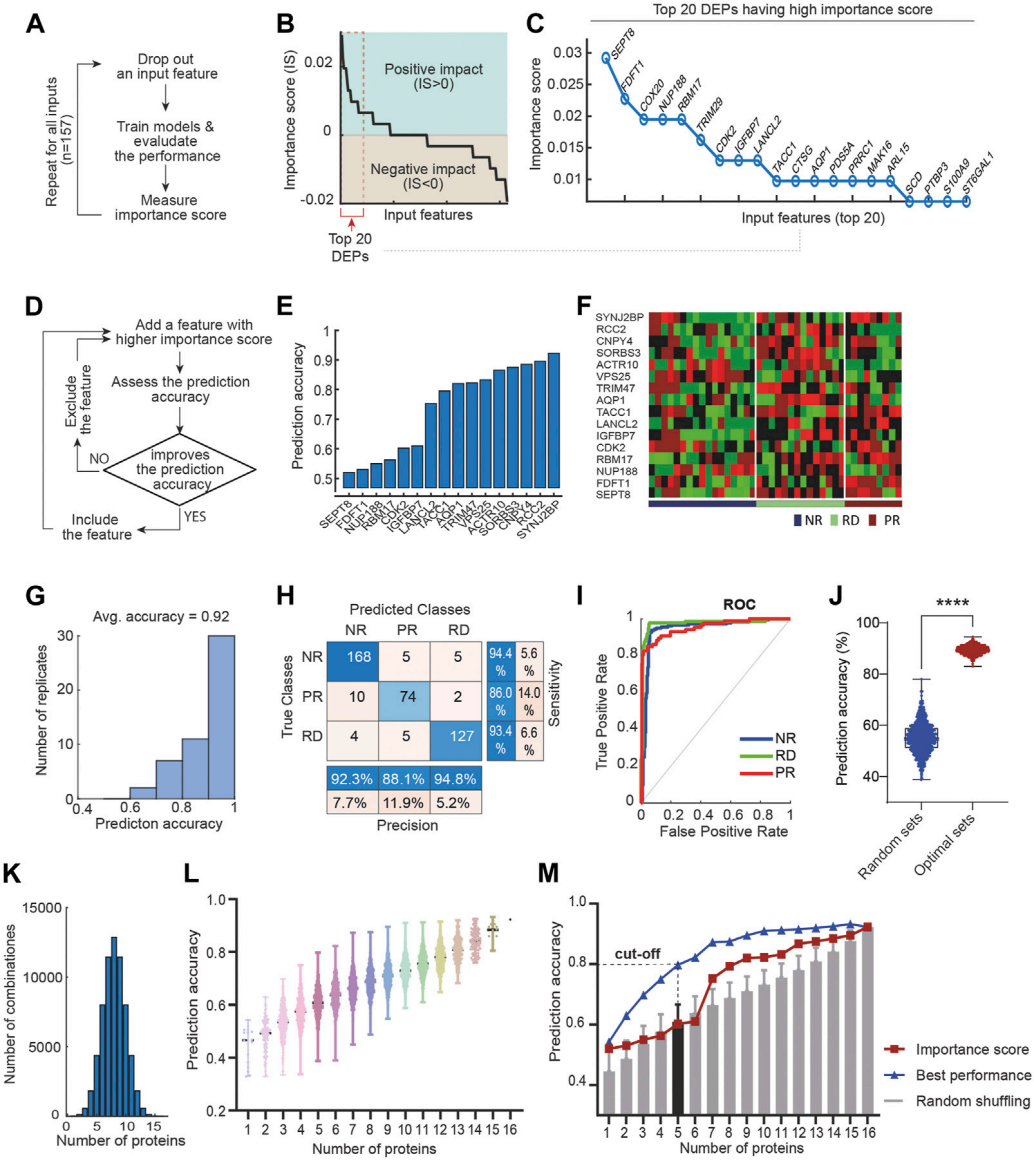
Rationalized feature selection optimizes ML model’s prediction accuracy. **(A)** A schematic of the drop-out analysis that enables calculation of the importance score (IS) for each feature. **(B)** Importance score values of all the DEPs, sorted in a descending order. IS > 0, <0, and = 0 indicates proteins having negative-impact, positive-impact and no-impact on drug response prediction, respectively. **(C)** Top 20 of 157 DEPs having the highest importance score values. **(D)** A schematic workflow of our IS-based feature selection strategy. **(E)** Gradual incorporation of 16 proteins that ultimately leads to optimal prediction accuracy of drug response. **(F)** Heatmap displaying the expression data of the identified 16 marker proteins across the discovery PDE cohort. **(G)** Distribution of prediction accuracy performance across 50 model replicate runs. **(H)** Performance of the optimal mSVM classifier using the 16 DEPs as input features, summarized in a confusion matrix. **(I)** ROC curves of the mSVM classifier for the RD, NR and PR response groups. **(J)** Comparison of performance between the optimal mSVM model and models using 16 randomly selected features (from 157 DEPs); **** *p*-value <0.0001 (unpaired *t*-test, *n* = 1,000). **(K)** Tally of possible combinations of proteins with increasing size ranging from 1 to 16. **(L)** Prediction accuracy significantly varies depending on the combination specification of input features. **(M)** Performance comparison between the optimal model and those with randomly selected features (shuffled from the 16 identified biomarkers), displayed for increasing panel size.

To select the most relevant features from the DEPs, we first performed a systematic feature drop-out analysis. One at a time, each DEP was removed from the feature space and the effect on performance of the mSVM was assessed, as compared to the original model using all the 157 DEPs as features (workflow in Figure 4A). If removal of a protein attenuates/improves the model prediction accuracy, then the protein is deemed to have a positive/negative impact on drug response prediction. We quantified these effects by defining an ‘importance score’ (IS) as in Eq. 1 that computes the difference in prediction accuracy between the drop-out and original mSVM models. Thus, IS > 0, <0, and = 0 indicates proteins having positive-impact, negative-impact and no-impact on drug response prediction, respectively (Materials and Methods). Figure 4B displays a sorted list of the 157 DEPs according to the respective IS values. Interestingly, a large fraction (48%) of the DEPs had a negative impact on drug response prediction (Figure 4B), suggesting inclusion of these in the feature space may diminish the model performance. In contrast, more than half of the DEPs had a positive impact on the drug response prediction (Figure 4B), with the top 20 proteins shown in Figure 4C.

We reasoned that the positive-impact DEPs would represent good candidate features for maximizing model prediction. We next introduced a new algorithm, termed greedy forward feature selection (GFFS), which aimed to select the optimal combination of features from the pool of positive-impact DEPs. A schematic of the algorithm is given in Figure 4D. First, we trained the mSVM using the positive-impact DEP having the highest IS (i.e., SEPT8; Figure 4E) as the single input variable, employing a similar training/validation data splitting scheme as in Section 3.2. Unsurprisingly, this single-feature model achieved ∼50% accuracy (Figure 4E), much worse compared to the model using all 157 DEPs. Next, we retrained the model by adding the second most influential DEP (i.e. FDFT1 having the second highest IS) to the feature space and re-evaluated the model performance. Because the new model had a better overall prediction accuracy, FDFT1 was kept as an input feature (Figure 4E). This process was repeated by gradually adding the next most important DEP to the feature space: if the new DEP improves prediction accuracy then it is kept; however, if it attenuates (or does not affect) accuracy, the protein is skipped and we move to the next positive-impact DEP. This was done until all the positive-impact DEPs were considered and the model performance did not further increase (Figure 4D). As a result, we determined an optimal feature space containing 16 DEPs, depicted in Figure 4E. The corresponding mSVM model achieved an overall prediction accuracy of 92% (Figures 4E–G), which was significantly superior to the initial model using all the 157 DEPs (77%, Figure 3B). This was further confirmed by examining the confusion matrix (Figure 4H) and the ROC curves (Figure 4I), indicating significantly improved prediction of 17-AGG response within each of the response groups.

A key attribute of our ML-based algorithm is the rationalized selection of features guided by prior IS-based ranking. To determine if this was critical in enhancing prediction accuracy, we assessed the performance of mSVM models using randomly selected features instead, and replicated this 1,000 times. The result shows that the model with feature selection consistently and significantly outperformed the random-feature models (Figure 4J), suggesting our IS-based feature selection strategy was key in boosting predictive power.

Next, we comparatively evaluated the performance of our IS-based GFFS approach with a range of available feature selection algorithms, including filter (ReliefF; minimum redundancy maximum relevance (MRMR)) (Ding and Peng, 2005; Stief et al., 2018), wrapper [recursive feature elimination (RFE); forward feature selection (FFS)] (Aha and Bankert, 1996; Tang et al., 2007; Marcano-Cedeño et al., 2010; Zhang et al., 2013) and embedded (boosting; bagging; least absolute shrinkage and selection operator (LASSO)) methods (Vasquez et al., 2016; Alsahaf et al., 2022) (Supplementary Figure S5A). GFFS showed significantly better predictive accuracy than all of the tested methods except for RFE, with which GFFS had comparable performance (Supplementary Figure S5A). Interestingly, the top three performers were GFFS, RFE, and FFS, highlighting the importance of rational feature selection in this context. We note that the maximal performance of FFS and RFE was achieved with 41 and 28 features, respectively (Supplementary Figures S5C, D), which were higher than GFFS, but at the cost of much larger number of features. Importantly, among of the top three methods, GFFS’s running time scales linearly and was significantly better than RFE and FFS (Supplementary Figure S5B). Thus overall, GFFS-based feature selection achieved a strong and balanced performance in terms of predictive accuracy and computational cost.

### Identification of a compact biomarker panel for 17-AAG treatment response

There is a general trade-off between the size of a biomarker panel and its practical applicability. A panel having more relevant proteins tends to deliver enhanced prediction, but this comes at a cost of having to detect more readouts from patients–a non-trivial task for poorly characterized biomarkers. In order to facilitate translation of the predictive biomarkers for 17-AAG-based therapy, here we aim to derive a more compact-size panel from the 16 identified marker proteins while maintaining high predictive performance. To this end, we considered all possible ways to combine the marker proteins into panels with increasing size, ranging from 1 to 16 (Figure 4K). As such, there are 16 possible panels with size 1; 120 panels with size 2; 4368 panels with size 5; and so on. We then evaluated the predictive performance of the mSVM model using each panel as input features. The results, displayed in Figure 4L, show that for each panel size the prediction accuracy varied significantly depending on the specific composition of the feature proteins (Supplementary Table S3). For instance, among 4368 5-protein panels, the one comprising AQP1, SEPT8, RBM17, TRIM47, and VPS25 exhibits the highest prediction accuracy of 80% (Figure 4L–M). Interestingly, this panel significantly outperformed the 5-protein panel derived from ranked IS score (accuracy 60%, Figure 4M), and panels derived from random shuffling (accuracy 61%, Figure 4M). Moreover, this 5-protein panel also outclassed the model using all the 157 DEPs (accuracy 77%, Figure 3B). Taken together, given its small size yet excellent predictive power, we concluded [AQP1, SEPT8, RBM17, TRIM47, VPS25] as a novel, practical biomarker panel for predicting response to 17-AAG treatment in prostate cancer.

Machine learning models have traditionally been treated as “black boxes”. As ML applications become more widespread, it is important to better interpret ML-based predictions and decision-making processes. Largely, the model interpretability (or explainability) methods can be categorized in two types: (i) global and (ii) local approaches (Ribeiro et al., 2016; Lundberg and Lee, 2017; Linardatos et al., 2021). Global explainability approaches explain the model’s behavior as a whole (across whole samples). For example, which features in the model contribute to the model’s prediction performance and how important they are. In Figure 4C, we have analysed the importance of individual features through performing a systematic “feature drop-out” analysis, which exactly corresponds to a global explainability method (Guidotti et al., 2019). On the other hands, local explainability approaches explain why and how the model make a particular decision for a particular sample (Guidotti et al., 2019). Among these, LIME (Local Interpretable Model-agnostic Explanations; (Ribeiro et al., 2016; Lundberg and Lee, 2017)] and SHAP (SHapley Additive exPlanations, (Lundberg and Lee, 2017; Linardatos et al., 2020)] have emerged as state-of-the-art approaches. For example, Gardiner et al. have recently applied SHAP to infer important features associated with drug responses (5-ASA, Prednisolone, BIRB796) for patients having inflammatory bowel diseases (Gardiner et al., 2022).

Thus, to examine the relative contribution of each feature (protein) to the prediction of drug response (RD, NR, and PR classification), we implemented SHAP (Lundberg and Lee, 2017; Linardatos et al., 2021) and LIME (Ribeiro et al., 2016; Lundberg and Lee, 2017) analyses. TRIM47, RBM17 and AQP1 were found to positively contribute to model prediction of the RD class; while the VPS25 and SEPT8 contribute negatively instead to the model prediction or not strong enough (Supplementary Figure S7A); The SHAP results were consistent with the importance score of LIME. On the other hand, RBM17 and AQP1 both have a positive impact on NR and PR classes but the contribution of TRIM47 is less significant for NR. The correlation analysis of features (proteins) with the Shapley values revealed that the AQP1 expression has a negative impact on the NR prediction but a positive impact on PR (Supplementary Table S4). VPS25 and SEPT8 showed a strong correlation with RD and NR although they did not contribute to the model prediction of target variables. Overall, these analyses helped enhance the interpretability of our ML model predictions.

### A Boolean algebra-based pipeline to derive biomarker expression signatures

**FIGURE 5:**
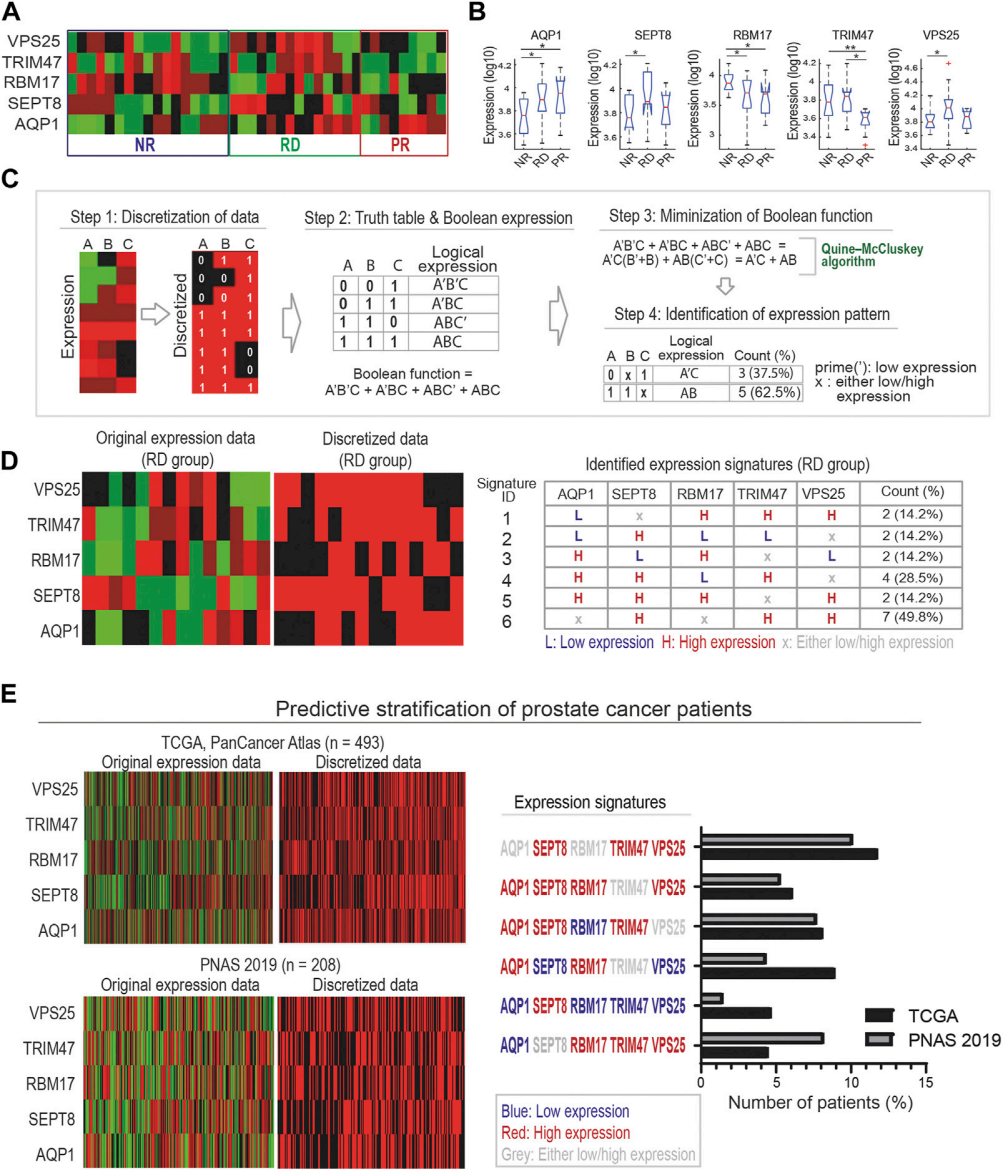
A Boolean algebra-based pipeline for derivation of biomarker expression signatures. **(A)** A heatmap displaying protein expression levels of the five marker proteins in our identified compact panel across the discovery PDE cohort. **(B)** Traditional statistical analyses using *t*-test and boxplot to compare expression levels of individual marker proteins between the response groups (* indicates *p*-value <0.05, ** <0.01 (unpaired *t*-test), a red sign indicates outlier data). **(C)** A multi-step Boolean algebra-based pipeline designed to identify combinatorial expression signatures of the biomarkers for each response group. Step 1: Discretization of protein expression levels into binary values. Step 2: Generation of truth table for binarized expression levels that are then transformed into Boolean expressions. Step 3: Minimization of Boolean functions using Quine-McCluskey algorithm, which converts it into simpler, more compact forms. Step 4: Identification of expression signatures of biomarkers. **(D)** Left: the original expression levels and corresponding binarized values of the five markers, shown for the RD group (left panels). Right: List of six identified expression signatures (ID 1–6) of the marker proteins, shown for the RD group. Similar data for the NR and PR groups is shown in Supplementary Figure S6. **(E)** Predictive stratification of prostate cancer patients, using two patient cohorts from the cBioPortal (Materials and Methods). Left: the original gene expression levels and corresponding binarized values of the five markers, shown for all the patients in each cohort. Right: number of patients identified with matching RD-specific expression signatures shown in Figure 5D.

Once the biomarkers have been identified, it is important to define specific expression signatures of these markers that could then be utilized for patient stratification. For this, analyses including *t*-test and boxplot are often employed to deduce the differential expression patterns of the marker proteins across the response groups. For example, the 17-AAG responsive PDEs (RD group) displayed significantly higher VPS25 expression, while those in the PR group have significantly lower TRIM47 expression compared to the other groups (Figures 5A, B). While useful, these approaches do not consider the expression heterogeneity within each response group (evidenced in Figure 5A) and possible hidden interlinks between the markers. Thus, derivation of biomarker signatures that encapsulate the response group-specific heterogeneity and possible functional links between the markers is important.

Here, we propose a new pipeline to identify combinatorial expression signatures for biomarkers characterizing individual response group utilizing methods from Boolean algebra. The pipeline consists of 4 steps and is illustrated in Figure 5C for example proteins A, B, and C. Step 1 discretizes the continuous expression data into binary values where 1 and 0 indicate high and low expression, respectively. This is done by normalizing the protein expression data to its median value across the samples: normalized value >1 or <1 will be converted to 1 or 0, respectively.

In step 2, all combinatorial binary expression patterns of the proteins are identified and summarized in a ‘truth’ table, which are then converted into logical expressions of the proteins (Figure 5C). Then, the logical expression of the individual patterns are summed together in a Sum-of-Products (SOP) form using the Boolean operator (+) (Materials and Methods) (Huntington, 1933). In step 3, the summed logical expression is reduced to a minimal form without losing information using a Boolean function minimization algorithm, the Quine-McCluskey algorithm (Jain et al., 2008). Finally, in step 4 the resulting reduced logical expression is converted back into binary expression patterns of the biomarkers. In the example in Figure 5C, we started with four expression patterns involving 3 proteins (A′B′C + A′BC + ABC’ + ABC) that were simplified into two patterns (A′C + AB) involving only 2 proteins (Hanf, 1975; Whitesitt, 2012) (see Figure 5C). Here, the prime (‘) sign indicates the respective protein should be low, and high otherwise.

Next, we applied the new pipeline to our previously identified 5-protein biomarker panel (Figure 5A). As a result, we identified six, five and four specific expression signatures of the biomarkers for the RD, NR and PR response groups, respectively (Figure 5D; Supplementary Figure S6). As an example, Figure 5D displays the six expression signatures for the RD group. Among these, signature ID 6, characterized by concomitant high expression of SEPT8, TRIM47, VPS25 while the expression of AQP1 and RBM17 could be either high or low, represents the most common signature among the RD-group PDEs (∼49.8%). The next most common signature, signature ID 4, is however characterized by high expression of AQP1, SEPT8, TRIM47 coupled with low expression of RBM17, while VPS25 expression could be high or low (Figure 5D). The biomarker signatures identified for the NR and PR groups are given in Supplementary Figure S6. In summary, our new Boolean logics-based pipeline has allowed us to identify specific expression signatures of the biomarkers that could be utilized to stratify patients for 17-AAG response.

### Validation of the biomarker signatures using independent PDE and patient cohorts

To demonstrate the utility of our derived biomarker signatures as a tool for patient stratification, we interrogated whether there are patients with matching 17-AAG-responsive signatures using publicly available prostate cancer patient datasets. To this end, two prostate cancer patient cohorts were obtained from the cBioPortal for Cancer Genomics database for analysis (see Materials and Methods). Patient-specific gene expression data of the 5 proteins in our biomarker panel were binarized as in step 1 of our pipeline (Figure 5E, left panels). Comparing the expression patterns of these proteins in the patients with the six identified signatures for the RD group showed that in both cohorts, a substantial fraction of the patients displays matching expression signatures (Figure 5E, right). Consistent with our PDE-based prediction, signature ID 6, the most frequent signature of the RD group (Figure 5D), was actually found in more patients than any other RD-specific signatures. Together, these findings support the utility of the derived biomarker signatures in identifying subsets of patients with specific drug response behaviour.

**FIGURE 6:**
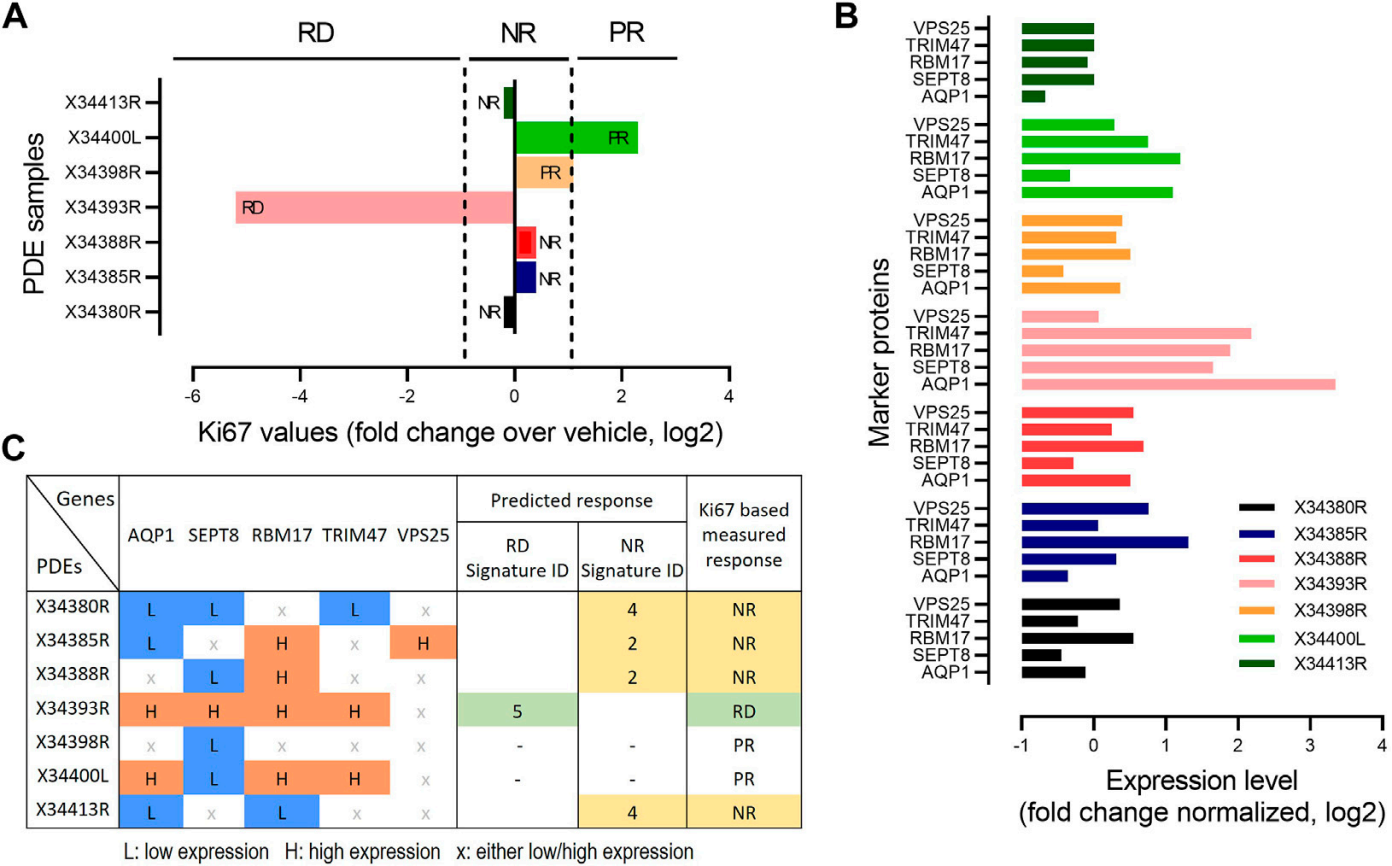
Validation of biomarker signatures. **(A)** Drug responsiveness of the seven PDEs to 17-AAG treatment based on fold change in *MKi-67* expression levels by the drug treatment relative to DMSO, measured by IHC. PDEs having > two-fold decrease in Ki-67 levels in response to the drug treatment compared to vehicle were defined as RD; having < two-fold increase as PR); otherwise, NR as in Figure 2B. **(B)** mRNA expression levels of the 5 genes in our identified compact biomarker panels measured by qRT-PCR for each PDE sample. The mRNA expression was normalized to GAPDH and TUBA1B. **(C)** Prediction of drug response based on the identified biomarker expression signatures, compared against Ki67-based response classification. The expression levels of the marker genes were binarized based on the median values by applying 30% and 70% quantile cut-offs: 0 indicates low expression (<30% quantile) and 1 indicates high expression (>70% quantile).

To further validate the predictive power of our identified biomarker signatures, we generated an independent cohort of prostate cancer patient derived explants (*n* = 7). Tissues were collected, cultured and analysed as previously described for our discovery cohort (Nguyen et al., 2018). These PDEs were treated with DMSO and 500 nM 17-AAG for 48 h. Treatment response to 17-AAG was assessed based on changes in Ki-67 positivity compared to vehicle treatment, detected using immunohistochemical staining and using similar cut-offs as done in the discovery cohort (Figure 6A, Materials and Methods). Baseline expression levels of the five biomarker genes AQP1, SEPT8, RBM17, TRIM47 and VPS25 were measured using qRT-PCR for each PDE under DMSO control (Figure 6B). Then, for each PDE we predicted the drug response using the expression signatures of the biomarkers identified using the Boolean optimization-based pipeline for the different response classes (Figure 5D; Supplementary Figure S6). Following the pipeline, for each PDE we first binarized the gene expression levels of the biomarkers into low or high expression, as shown in Figure 6C. The biomarker expression patterns for each PDE were then mapped to the identified signatures for the three response groups. For example, the expression pattern for PDE X34393R matches with Signature ID 5 of the RD group, which correctly predicted this PDE to be responsive to 17-AAG. On the other hand, PDE X34380R matches with Signature ID4 of the NR group, thus correctly predicting this PDE to be non-responsive to the drug. Overall, cross-validating model predictions with measured drug response, our identified signatures correctly predicted response classification for the responsive and non-responsive PDEs, but did not correctly predict the poor-responders, achieving an overall >71% accuracy on this independent dataset. Despite the small size of the validation cohort, this independent validation analysis has provided a proof-of-concept demonstrating the potential of our predictive pipeline. We envisage as more similar data become available in the future, further validation will be done to strengthen the validity of the identified biomarkers.

## Discussion

Precision oncology embraces cancer treatment strategies that are based on the distinct molecular characteristics of a tumour. However, lack of predictive companion biomarkers that help forecast patient-specific treatment response remains a barrier to widespread adoption of this paradigm (Mateo et al., 2022; Pich et al., 2022). In this study, we have developed a novel computational framework that couples supervised machine learning-based biomarker discovery with Boolean algebra-based signature derivation, in order to identify predictive multi-gene biomarker signatures for cancer therapies (Figure 1). We demonstrated the utility of the approach by applying it to the HSP90 inhibitor 17-AAG in the context of prostate cancer. The approach is however broadly applicable, and given suitable data, can be deployed for different drugs in various tumour types.

The new framework possesses two salient distinguishing properties. First, it rationalizes the most predictive input features based on an importance score that measures how each feature influences the model’s predictive performance. Only features that contribute positively to the classification accuracy are retained in the feature space. These are then ranked by their IS values, and increasingly combined one-by-one to identify the optimal combinatorial panel of features that delivers the maximal predictive accuracy. Second, once the biomarkers have been identified, our framework innovatively utilizes Boolean algebra and function minimization [Quine-McCluskey algorithm (Quine, 1955; McCluskey, 1956)] techniques to deduce common expression patterns of the response-specific biomarkers. Because Quine-McCluskey algorithm enables the minimal form of a Boolean function to be reached, our framework helps derive the most compact response group-specific biomarker expression patterns. These easily-interpretable patterns thus constitute biomarker signatures that ultimately allows predictive selection of patient subgroups having a particular drug response, an ability invaluable for precision clinical trials and treatment.

Boolean function minimization algorithms aim to identify the core logics of the underlying phenomenon and are routinely used in engineering fields, such as to design digital logic circuits (Huntington, 1933; Jain et al., 2008). We have previously applied Boolean function minimization to identify core combinatorial feedback loop structures that generate switch-like behavior of E-cadherin (Shin et al., 2010). To our best knowledge, the current study represents the first attempt to apply Boolean function minimization to the problem of biomarker signature derivation. Nevertheless, Boolean logics-based approaches have been used to predict drug response. For example, the LOBICO (Logic Optimization for Binary Input to Continuous Output) modelling framework was developed to explain drug response in cancer cell lines based on binary mutation data of 60 selected genes (Knijnenburg et al., 2016). Using integer linear programming, LOBICO aims to identify the logic combinations of mutations that best explain the response of cancer cell lines to cancer drug agents. In a similar vein, MOCA (Multivariate Organization of Combinatorial Alterations) has been applied to predict drug response by inferring logic combinations of genomic input features (Masica and Karchin, 2013). Overall, our framework represents a novel effort in repurposing Boolean function minimization techniques for derivation of drug-response biomarker signatures.

The results in this study emphasize the importance of rational feature selection in optimizing drug response prediction accuracy by machine learning classifiers. While our GFFS approach is similar to FFS in the sense that it starts with no feature, it differs in two key aspects. Firstly, it pre-determines the relative importance of the input features by calculating the IS beforehand *via* comparison of model performance, and it does this only once (Figures 4A–C). This is opposed to the classical implementation of FFS, where the relative importance ranking of the remaining features is repeatedly evaluated at each iteration. Secondly, GFFS goes through the pre-ranked features, adds each feature and keeps the feature only if it improves the model performance; otherwise, the feature is dropped and the algorithm moves on to the next feature in the ranked list. Again, this differs from the classical FFS, where addition of a new best feature (n+1 features) at the current round may reduce the overall model performance as compared to the optimal model at the previous round (n features), as shown in Fig. S5C. This difference stems from the fact that in FFS-based implementation, a specific number of features is typically specified prior to model running whereas GFFS does not require such specification. Another consequential difference is that the model performance increases monotonically as more features are added with our approach (Figure 4E), while for FFS the model performance could exhibit a drop as new features are added (Supplementary Figure S5C). Importantly, because of the pre-determined feature ranking, GFFS is computationally much more efficient than FFS and RF. In Big-O notation, our algorithm has O (2n) complexity compared to O (n (n+1)/2) complexity displayed by RFE/FFS (see Supplementary Figure S5B). This superiority in computational cost makes GFFS highly scalable compared to other feature selection techniques, particularly when the number of input features to be assessed is in the range of thousands to tens of thousands.

It is worthy to note that in our two-phase framework, the machine learning coupled GFFS feature selection (phase 1) is integrated with but can work independently from the Boolean logics-based biomarker signature identification (phase 2). As such, in principle the Boolean logics-based biomarker signature identification part can be plugged into any other feature section ML approaches (e.g., using FFS or RFE) and serves as a downstream analysis. This plug-and-play flexibility provides another strength of the framework.

Due to its ability to stabilize client oncogenic proteins and thereby maintain the survival of cancer cells, HSP90 presents an attractive therapeutic target and has been explored in a variety of cancers including prostate, breast, and colon cancer (Caldas-Lopes et al., 2009; Wang et al., 2016; Nguyen et al., 2018). Although limited, several studies have attempted to identify predictive markers for HSP90-based therapy. For example, Nguyen et al. (Nguyen et al., 2018) has identified PCBP3, an RNA binding protein important in post-transcriptional control of gene expression, as a potential predictive biomarker for 17-AAG response in prostate cancer. In colorectal cancer, high expression of the UDP glucuronosyltransferase 1A (UGT1A) gene was found to correlate with poor sensitivities to the HSP90 inhibitor ganetespib, and its related compound NVP-AUY922, suggesting UGT1A levels in tumour tissues may be a suitable predictive biomarker for ganetespib treatment (Landmann et al., 2014). Interestingly, gene expression levels of UGT1A did not show correlation with 17-AAG response, implying different classes of HSP90 inhibitors may have different predictive biomarkers (Landmann et al., 2014). In addition, in acute lymphoblastic leukemia (ALL), patients with high levels of phosphorylated Src were more sensitive to the Hsp90 inhibitor NVP-BEP800 compared to those with low phosphorylated Src (Mshaik et al., 2021), suggesting Src phosphorylation may serve as a predictive biomarker. Moreover, since Hsp90 inhibition regulates Akt phosphorylation and Bcl-xL, expression levels of these effector proteins may be suitable predictive of response to Hsp90 inhibition in triple negative breast cancer (Caldas-Lopes et al., 2009). Similarly, as Hsp90 inhibition downregulates c-Myc expression and upregulates the expression of tumour repressor proteins such as p53 and pRB, which inhibits the G1/S transition (Yamaki et al., 2011), expression levels of cell cycle regulatory proteins such as pRB, E2F, cyclin–cyclin-dependent kinase (CDK) complexes could inform predictive biomarkers in specific tumour contexts. However, there are several limitations associated with current studies of predictive biomarkers for Hsp90 inhibitors, including: (i) their derivation was largely based on correlation analyses; (ii) the biomarkers are mostly single-gene markers and so unlikely to be clinically robust; (iii) and lack of patient-derived data. Together, these factors may explain the fact that so far, no companion predictive biomarkers of Hsp90-based therapy are employed for clinical practice.

In this study, we have aimed to alleviate these limitations through utilization of patient-derived data from unique explant models; implementation of predictive ML modelling rather than association analyses; and derivation of multi-gene rather than single-gene biomarkers. As a result, we have identified a highly-predictive biomarker panel (92% accuracy) consisting of 16 proteins. Its superior performance to individual DEPs and to using all the 157 DEPs points to the importance of selectively combining the relevant input features in optimizing drug-response prediction. The result also highlights the need to venture beyond the contemporary single-marker paradigm. Reassuringly, the identified panel contains proteins that have been implicated in prostate tumorigenesis and drug resistance, including CDK2, IGFBP7, TRIM47, and RBM17. For example, CDK2 was identified as a therapeutic target in prostate cancer (Yin et al., 2018). Its activation is significantly associated with disease recurrence, and its inhibition reduces invasion of prostate cancer cell lines (Yin et al., 2018). Moreover, CDK2 mediates androgen-dependent inhibition of AR+, castration-resistant prostate cancer cell proliferation (Kokontis et al., 2014). IGFBP7, a member of the insulin growth factor binding protein family, is involved in a variety of cancers including prostate cancer (Sullivan et al., 2012; Jin et al., 2020). Aberrant promoter hypermethylation of IGFBP7 and consequential gene silencing were found in prostate cancer cell lines (Sullivan et al., 2012). On the other hand, the tripartite motif (TRIM) protein TRIM47 is significantly increased in prostate cancer compared to normal tissues (Fujimura et al., 2016). In addition, SPF45, a splicing factor, is overexpressed in select tumours including prostate cancer, and it confers resistance to multiple anti-cancer drugs (Sampath et al., 2003; Perry et al., 2005). Overall, these evidences support the validity of our predictive multi-protein biomarker panel.

Translation of predictive biomarkers into clinical usage depends strongly on the ability to develop assays for detection of these markers in patient samples. We therefore reasoned that compact biomarker panels displaying strong predictive power are optimal for clinical application. With this in mind, we reduced the panel from 16 to 5 proteins, which achieved excellent prediction accuracy (80%). The 5-protein panel includes VPS25 (Vacuolar Protein Sorting 25 Homolog), TRIM47 (Tripartite motif 47), RBM17 (RNA Binding Motif Protein 17), SEPT8 (Septin-8) and AQP1 (Aquaporin 1). In addition to TRIM47’s involvement in prostate cancer mentioned above, RBM17 is frequently overexpressed in a variety of carcinomas, including prostate cancer (Sampath et al., 2003). Importantly, RBM17 confers resistance to doxorubicin and vincristine, two chemotherapeutic drugs commonly used in cancer treatment (Perry et al., 2005). Septins are GTP-binding proteins that are evolutionarily and structurally related to the *RAS* oncogenes (Abbey et al., 2019). Septin’s expression levels are altered in hormonally regulated cancers such as prostate, breast, ovarian and endometrial cancers (Dolat et al., 2014; Angelis and Spiliotis, 2016). AQP1 is known to be upregulated by hypoxia that leads to increased cell water permeability, motility, and migration in neuroblastoma, lung and prostate cancer cells (Mobasheri et al., 2005; Hwang et al., 2012; Wei and Dong, 2015; Huo et al., 2021). Further, AQP1 is involved in microvascular alteration during prostate tumour angiogenesis (Mobasheri et al., 2005); and it promotes sensitivity of anthracycline chemotherapy in breast cancer (Chong et al., 2021). Taken together, these studies provide evidence linking the identified marker proteins to prostate cancer, supporting to the validity of the simplified panel. Further understanding of the roles of these proteins in prostate cancer tumorigenesis, and how they mechanistically modulate 17-AAG sensitivity are important areas of future research.

In addition, we conducted KEGG pathway and GO function analysis using both the 5-protein and 16-protein biomarker panels. As shown in the Supplementary Figure S8, the 5-proteins panel is mainly related to the proximal tubule bicarbonate reclamation (Dubose, 1990) and renin secretion (Kurtz, 2012) in the KEGG pathway analysis. Proximal tubule bicarbonate reclamation is a process by which the proximal tubules in the kidney reclaim bicarbonate ions from the filtrate in the renal tubules (Rector et al., 1998). This process helps to maintain electrolyte balance in the body by reabsorbing bicarbonate ions and preventing their excretion in urine. Renin secretion is important in cancer development as it regulates the production of angiotensin II, which has been shown to stimulate cancer cell growth and proliferation (Sobczuk et al., 2017). The 16-protein panel was found to be mainly related to steroid biosynthesis, a process by which the body produces steroid hormones. Abnormal steroid hormone production, which can be influenced by abnormalities in steroid biosynthesis pathways, has been linked to prostate cancer (Wilding, 1992; Mostaghel, 2013).

Our GO function analysis identified that both the 5- and 16-protein biomarker panels are mainly related to polyol transmembrane transporter activity (GO:0015166) and intracellular cGMP-activated cation channel activity (GO:0005223). Polyol transmembrane transporter activity involves the transport of small sugar molecules, such as glucose, across cell membranes. Dysregulation of this activity has been implicated in cancer development, as it can contribute to increased cellular proliferation and survival (Jones and Morris, 2016). Intracellular cGMP-activated cation channels are proteins activated by the signaling molecule cGMP, which allow ions to enter cells (Biel and Michalakis, 2009). Dysregulation of these channels has been linked to the development of various types of cancer, including breast, prostate, and ovarian cancer (Di Iorio et al., 2021).

Importantly, in an effort to validate the identified 5-protein biomarker signatures, we have generated an independent validation cohort of PDEs, and predicted their responses to 17-AAG treatment based on the PDE-specific expression levels of the five marker proteins. Overall, our framework correctly predicted the response for the responsive and non-responsive PDEs, but did not correctly predict the poor-responders, achieving >71% accuracy on this independent dataset. A limitation of the current validation is pertained to the small size of the validation cohort, due primarily to the challenge in accessing a large number of suitable patient samples and establishing the corresponding PDEs. This, however, is a general issue in biomarker studies utilizing pharmacogenomic data derived from cancer patients (Huang et al., 2018; Parca et al., 2019; Nguyen et al., 2021). We envisage as additional PDEs are generated in the future, the data will provide a more robust validation of our pipeline.

In summary, we have developed a new computational framework based on machine learning that aids the identification of multi-gene predictive biomarkers for targeted cancer drugs. While we have demonstrated its power focusing on prostate cancer as a proof-of-concept, the framework has broad applicability and can be applied to other drugs and cancer types in future studies.

## Data Availability

The datasets presented in this study can be found in online repositories. The names of the repository/repositories and accession number(s) can be found below: TCGA, cancer_study_identifier: prad_tcga_pan_can_atlas_2018, PNAS cancer_study_identifier: prad_su2c_2019.
