# Supplementary material for: A Boolean-based machine learning framework identifies predictive biomarkers of HSP90-targeted therapy response in prostate cancer
Source: Front Mol Biosci. 2023 Jan 19;10:1094321. doi: 10.3389/fmolb.2023.1094321 (PMC9892654; doi:10.3389/fmolb.2023.1094321)
Supplement: Supplementary file 4 [file DataSheet1.DOCX]

A Boolean-based machine learning framework identifies predictive biomarkers of HSP90-targeted therapy response in prostate cancer

Supplementary Material

# Supplementary Figures and Tables

## Supplementary Figures

**
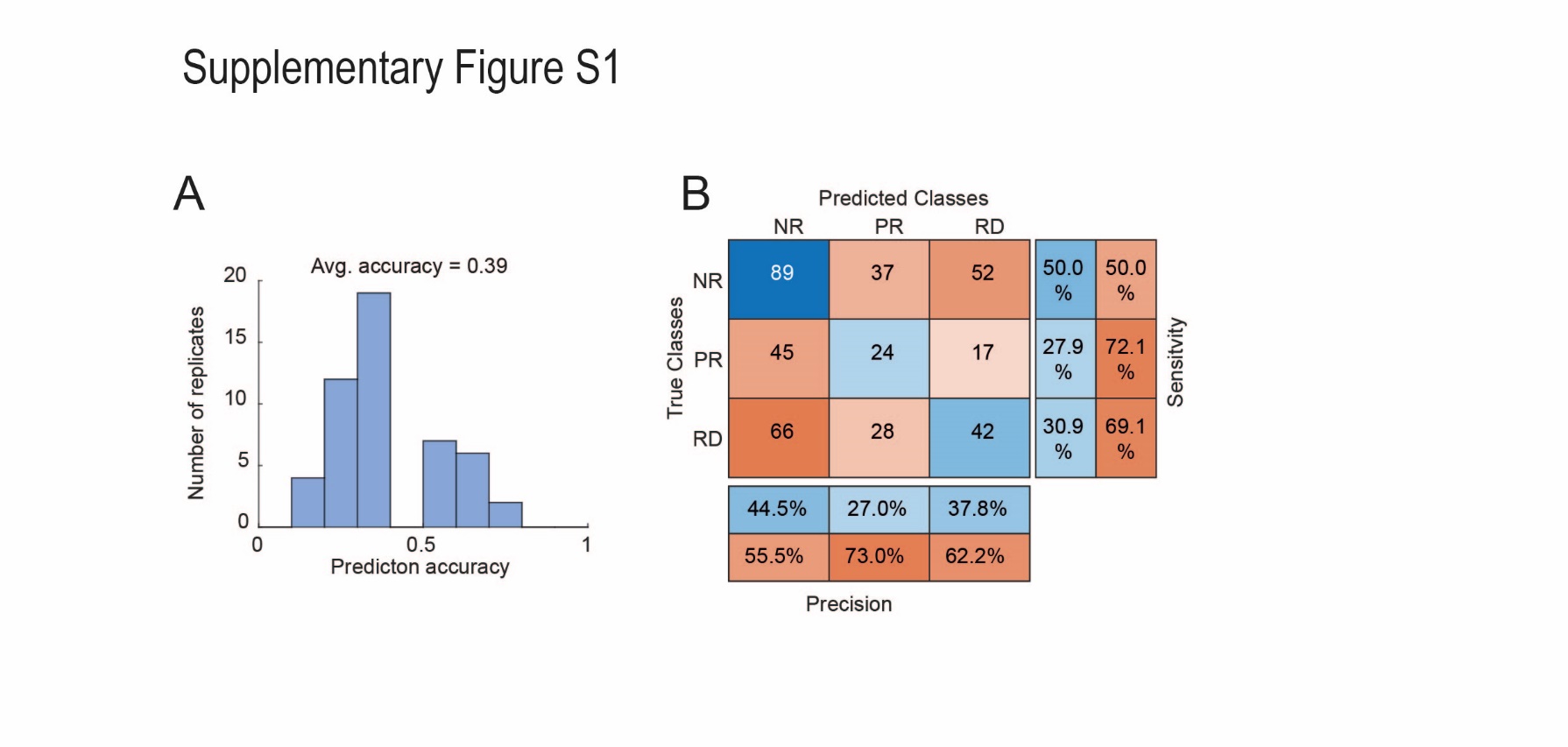
Supplementary Figure S1.** **(A)** Distribution of the prediction accuracy (number of cross-validation = 50). **(B)** The confusion matrix.


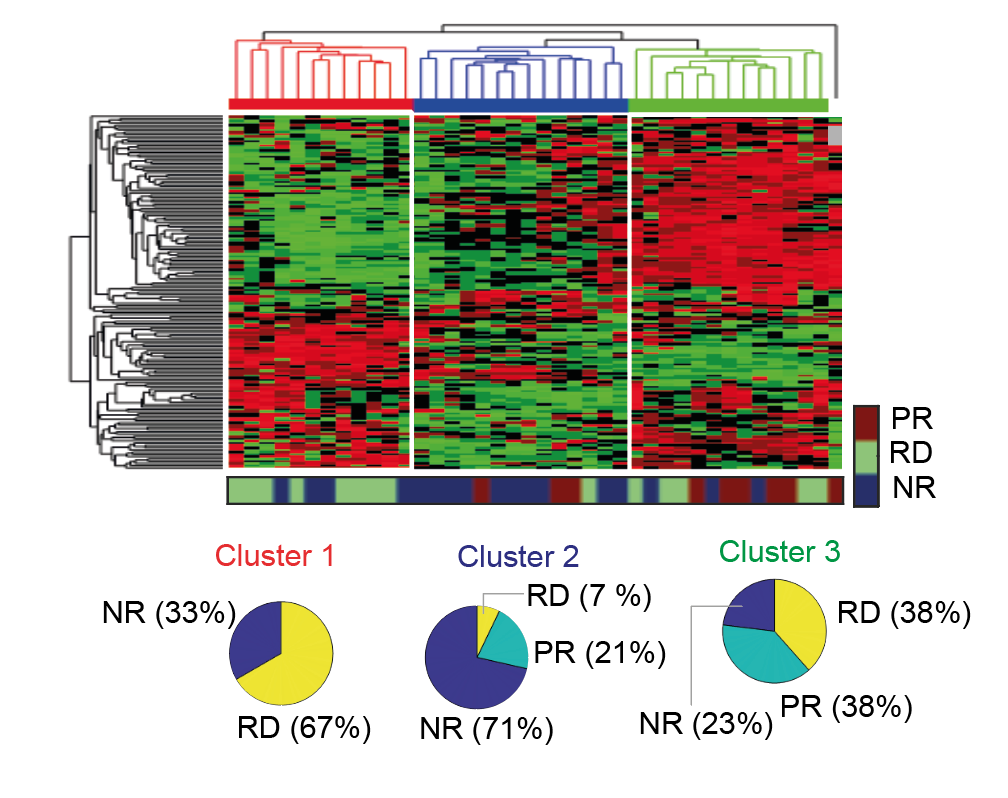


**Supplementary Figure S2.** Unsupervised hierarchical clustering of response groups of PDEs which failed to predict response to Hsp90 inhibitor 17-AAG.


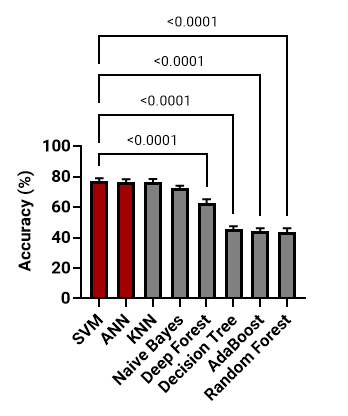


**Supplementary Figure S3.** **Comparison of model prediction performance of various machine learning algorithms**. The p-values were calculated using unpaired t-test (n=50). SVM: Support Vector Machine ANN: Artificial Neural Network, KNN: K-Nearest Neighbor.


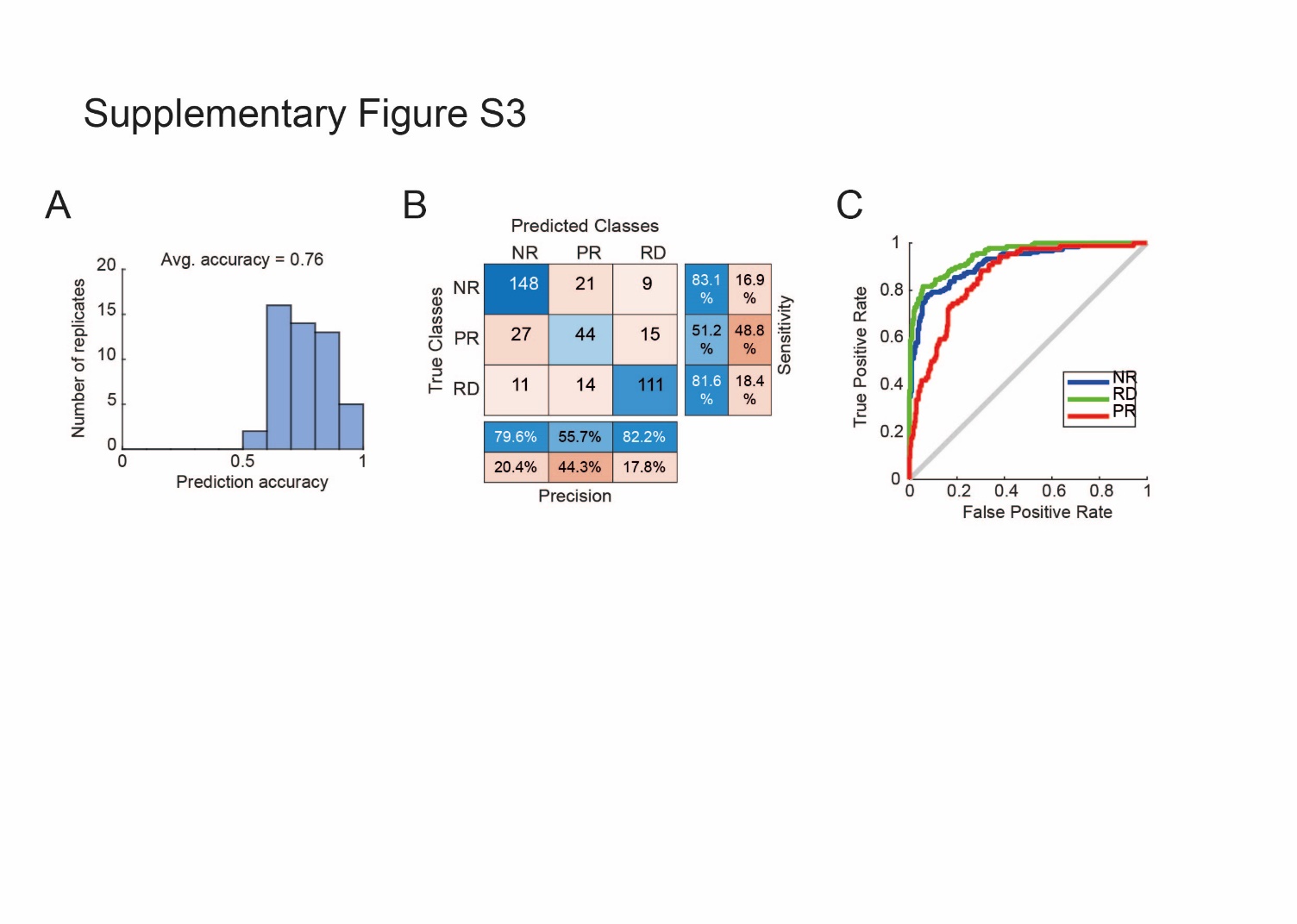
**Supplementary Figure S4. Prediction of drug responses using DEPs based on ANN.** **(A)** Distribution of the prediction accuracy (number of cross-validation = 50). **(B)** The confusion matrix. **(C)** ROC curves of RD, NR and PR classes.


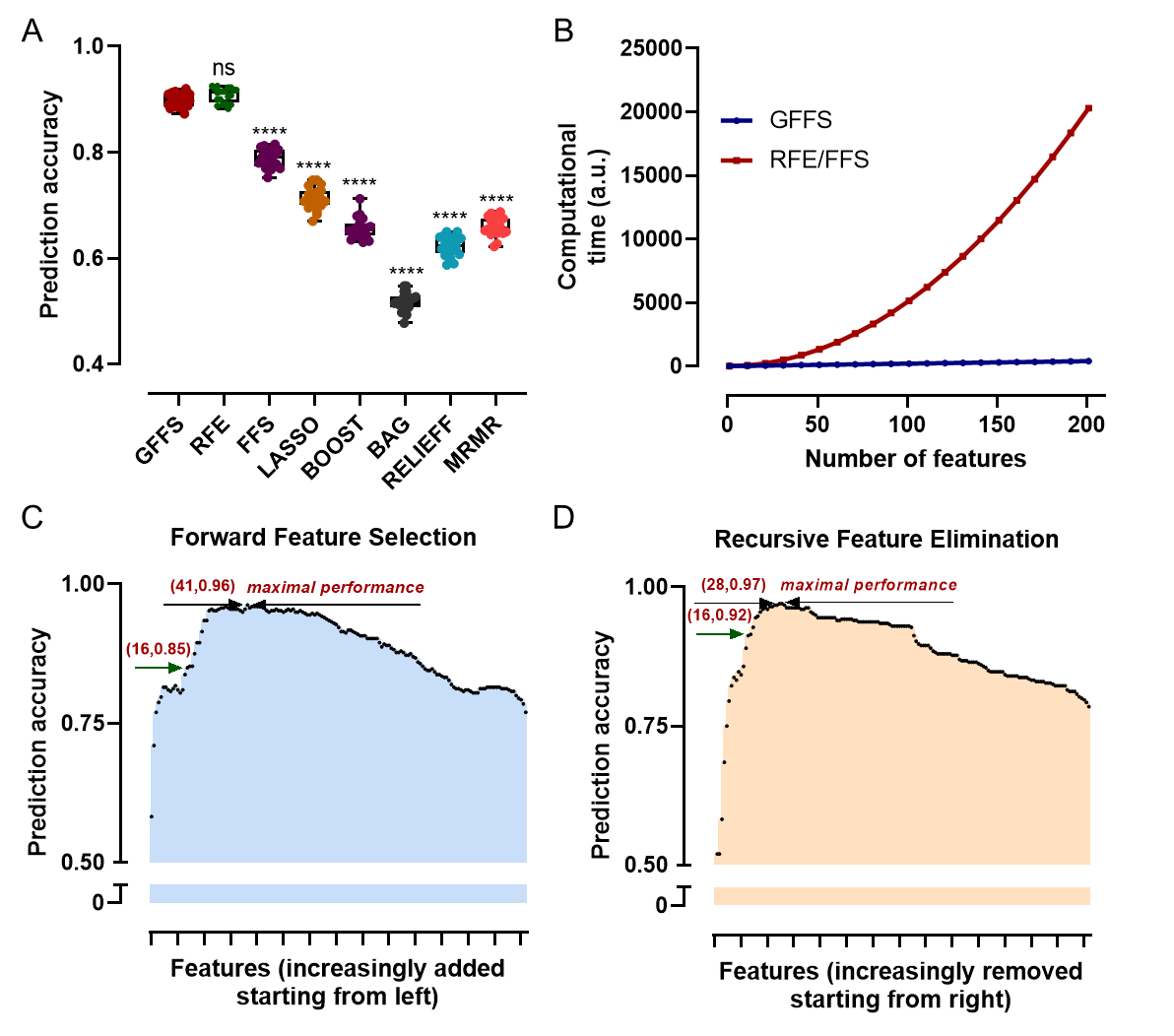
 **Supplementary Figure S5. Comparative analysis of various feature selection strategies.** **(A)** Comparison of predictive performance between different feature selection models. GFFS: greedy forward feature selection (our method); RFE: recursive feature elimination; FFS: forward feature selection; LASSO: least absolute shrinkage selector operator; BOOST: adaptive boosting ensemble method; BAG: bagging ensemble method; RELIEFF: ReliefF algorithm; MRMR: minimum redundancy maximum relevance algorithm. ns: no significant, **** indicate p-value < 0.0001 (unpaired t-test, n=20 independent repeats with random partitioning) as compared to GFFS. **(B)** Comparison of computational running time between GFFS and FFS/RFE. **(C-D)** Model predictive performance curves of FFS (C) and RFE (D) at different number of selected features. The green arrows indicate model performance for 16 selected features, and the blue arrows indicate the maximal performance with the associated number of selected features.


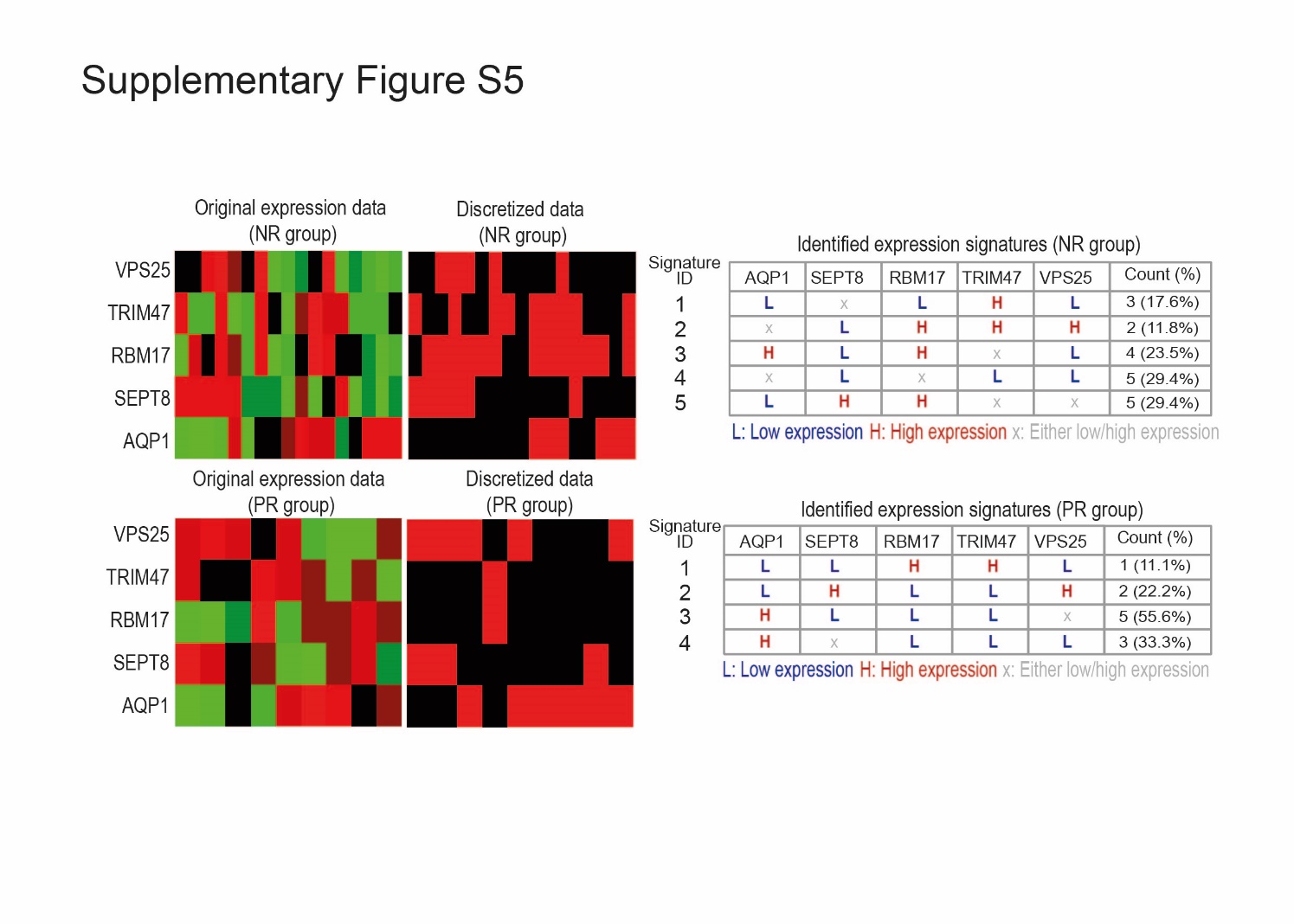


**Supplementary Figure S6. Expression signature of the predictive maker proteins.** The expression and discretized data of the predictive five marker genes in NR and PD groups of PDE samples (left panels). The expression signatures of the marker proteins (right panel) identified from applying Boolean function minimization algorithm. L and H indicates low and high expression of protein respectively. x denotes either low or high expression.


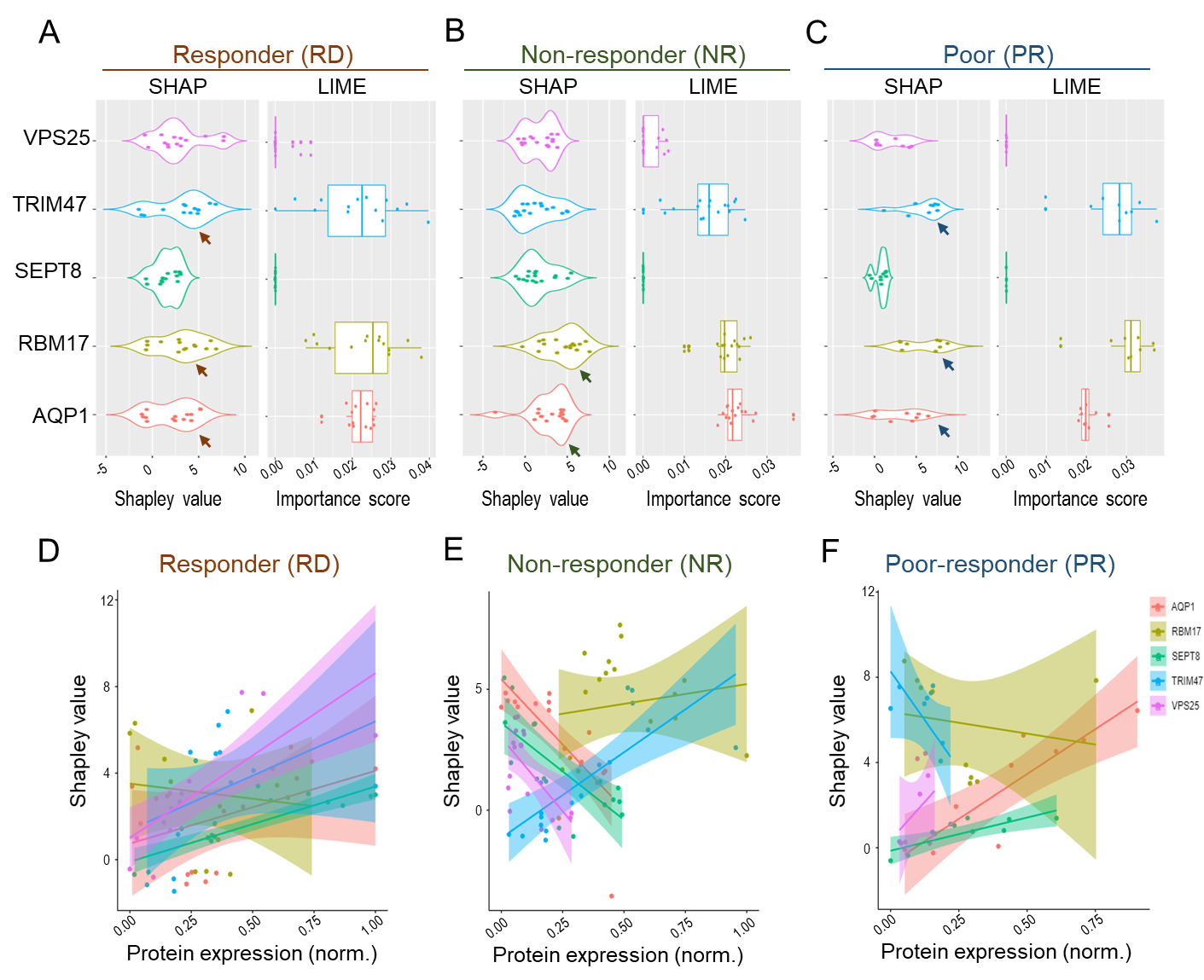


**Supplementary Figure S7**. **Analysis of relative contribution of features to model prediction** using interpretable ML methods: SHAP and LIME **(A-C)**. Correlation analysis of features (proteins) with the target variables (drug responses) **(D-F)**. The dots indicate the train data. The line and shade are a linear regress of data and its confidence interval, respectively. Shapley values and the importance scores (LIME) of the features were calculated against all the training dataset (n=40).


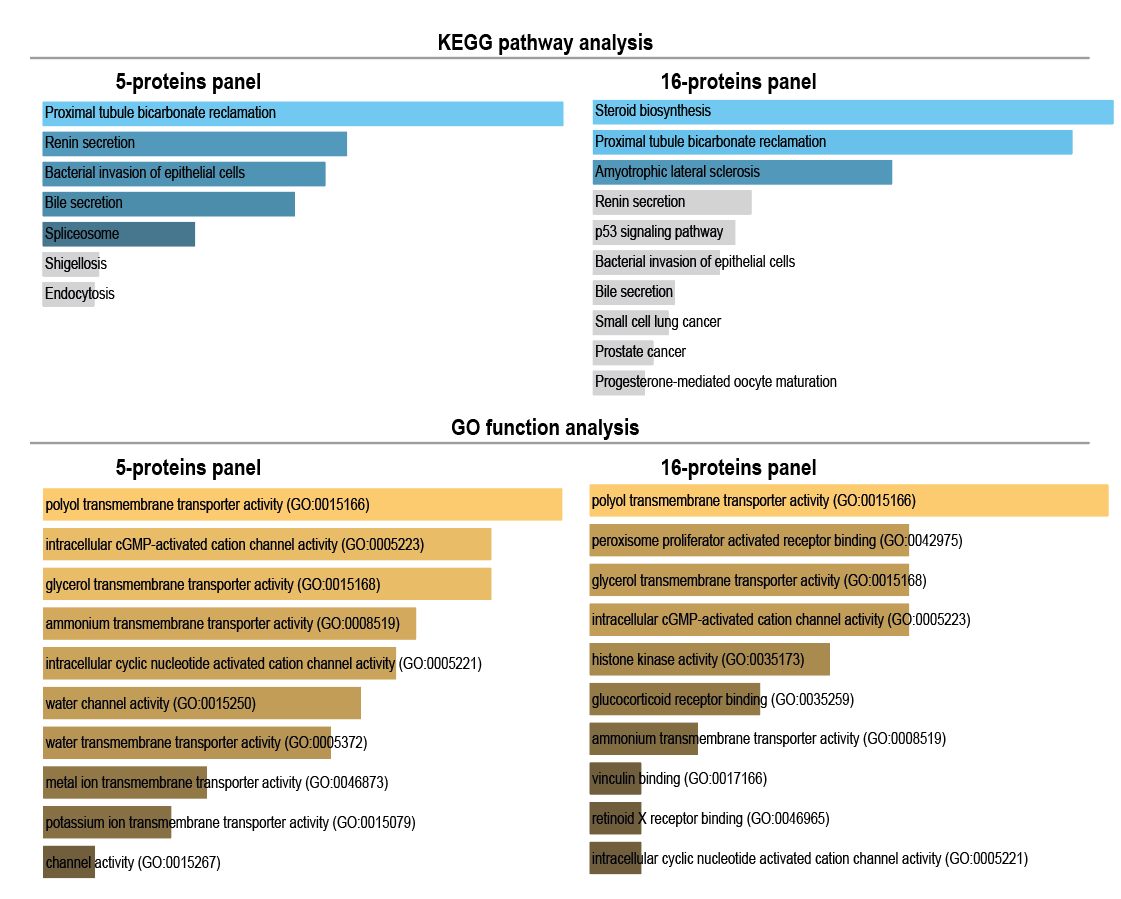


**Supplementary** **Figure S8. KEGGF pathway and GO function analysis using the 5 and 16-protein biomarker panels.** Note that the grey color indicates no statistical significance.

## Supplementary Tables

**Supplementary Table S1. Classification of the PDE samples into three response groups based on the corresponding Ki67 positivity upon treatment with 17-AAG.**

**Supplementary Table S2. 157 differentially expressed proteins (DEPs) obtained from differential expression analysis between 3 response groups (ANOVA, p value < 0.05)**

**Supplementary Table S3. Combination of the 16 optimal input features and prediction capacity**

**Supplementary Table S4. Correlation analysis of features and target variables (see Figure S6D-F)**

|  | RD | | NR | | PR | |
| --- | --- | --- | --- | --- | --- | --- |
|  | correlation  coefficient | P-value | correlation  coefficient | P-value | correlation  coefficient | P-value |
| AQP1 | 0.37 | 0.19 | -0.79 | 0.00016 | 0.88 | 0.0018 |
| RBM17 | -0.13 | 0.66 | 0.15 | 0.26 | -0.18 | 0.65 |
| SEPT8 | 0.91 | 6.40E-06 | -0.73 | 0.00085 | 0.81 | 0.0077 |
| TRIM47 | 0.41 | 0.14 | 0.78 | 0.00025 | -0.61 | 0.08 |
| VPS25 | 0.77 | 0.0013 | -0.61 | 0.0097 | 0.38 | 0.32 |

**Supplementary Table S5. Primer sequences used for the qPCRs**

| **Gene** | **Primer name** | **Sequence (5' - 3')** |
| --- | --- | --- |
| MKI67 | MKI67-F | GCCTGCTCGACCCTACAGA |
|  | MKI67-R | GCTTGTCAACTGCGGTTGC |
| AQP1 | AQP1-F | GGACACCTCCTGGCTATTGA |
|  | AQP1-R | TCCAGTGGTTGCTGAAGTTG |
| RBM17 | RBM17-F | TTTGCAAGGAGACCAGATCC |
|  | RBM17-R | AGCTCCGCCCATACTTCTTT |
| TRIM47 | TRIM47-F | ACAGAAGCTGGACTCGGAAG |
|  | TRIM47-R | TCCCTGGGAGCTTCACTCT |
| VPS25 | VPS25-F | TCCAGCTTCCTGATCATGTG |
|  | VPS25-R | AGTTGTTCTGGCCACTCCTG |
| SEPT8 | SEPTIN8-F | GAAAGGGAGCTCCATGAGAA |
|  | SEPTIN8-R | ATTGAAGGCGTTGGTCTCCT |
| GAPDH | GAPDH-F | TGCACCACCAACTGCTTAGC |
|  | GAPDH-R | GGCATGGACTGTGGTCATGAG |
| TUBA1B | TUBA1B-F | CCTTCGCCTCCTAATCCCTA |
|  | TUBA1B-R | CCGTGTTCCAGGCAGTAGA |
